# Evaluation of a Novel Goals-of-Care Discussion Priming Tool (MyCare) in Inpatient General Internal Medicine Ward Settings: Feasibility, Acceptability, and Usability Study

**DOI:** 10.2196/66932

**Published:** 2025-10-28

**Authors:** Maren Kimura, Sydney Ruller, Alan Forster, James Downar, Sarina R Isenberg, Leah Steinberg, Kumanan Wilson, Victoria Shaffer, Pete Wegier, Jeff Myers, Russell Goldman, Carolyn Steele Gray, Daniel Kobewka

**Affiliations:** 1 Ottawa Hospital Research Institute Ottawa, ON Canada; 2 Division of Palliative Care The Ottawa Hospital Ottawa, ON Canada; 3 Bruyère Research Institute Ottawa, ON Canada; 4 Department of Medicine University of Ottawa Ottawa, ON Canada; 5 Temmy Latner Centre for Palliative Care Sinai Health System Toronto, ON Canada; 6 Division of General Internal Medicine Department of Medicine The Ottawa Hospital Ottawa, ON Canada; 7 Department of Psychological Sciences University of Missouri Columbia, MO United States; 8 Science of Care Institute Lunenfeld-Tanenbaum Research Institute Sinai Health Toronto, ON Canada; 9 Institute of Health Policy Management and Evaluation University of Toronto Toronto, ON Canada

**Keywords:** goals of care, web-based tool, patient conversations, mixed methods, inpatient, medicine ward, medicine, ward, prognosis, person-centered care, pilot study, feasibility, acceptability, usability, ill patient, semistructured interviews, communication, web-based, tool, doctor-patient communication

## Abstract

**Background:**

For patients who are seriously ill, conversations about prognosis, values, and goals can improve person-centered care; however, these conversations do not occur consistently.

**Objective:**

We aimed to develop an online, interactive goals-of-care discussion priming tool called MyCare to facilitate conversations about patient goals among physicians, patients, and family members. Our objective was to determine the feasibility, acceptability, and usability of MyCare for patients who are seriously ill in hospitals.

**Methods:**

We conducted a mixed methods study on internal medicine wards with patients who were seriously ill at 2 hospitals. Participants completed MyCare with a research assistant in their hospital room, and their responses were sent to their attending physician team. Patients, family members, and physicians participated in semistructured interviews to assess advance care planning engagement, usability, and acceptability. Patients also completed the System Usability Scale, the eHealth literacy scale, and survey questions about acceptability.

**Results:**

Patients took a median time of 32 (IQR 21-43) minutes to complete MyCare, with a range of 12 to 101 minutes. The mean eHealth literacy score was 56 out of 100. In total, 19 (76%) out of 25 patients indicated that MyCare helped them think about and understand what was most important to them, and 16 (64%) stated that MyCare helped them think about the type of care they would want if their illness progressed. The mean advance care planning engagement scores on a 5-point scale before and after MyCare were 4.11 (SD 0.77) and 4.20 (SD 0.74), respectively (mean difference 0.09, 95% CI –0.08 to 0.34; *P*=.22). After completing MyCare, 16% (4/25) of the patients reported feeling more prepared to talk to their medical decision-maker about the type of care they would want if they were very sick or near the end of life, and none felt less prepared. The most useful elements for patients were (1) clarifying goals and wishes for care, (2) identifying people who supported them, and (3) having a resource to facilitate conversations with their families. The most useful elements for physicians were patients’ prioritized values and goals.

**Conclusions:**

MyCare and similar tools will be more usable and acceptable if they are short and simple, focus on shared physician-patient priorities, and empower patients to advocate for their own goals.

## Introduction

### Background

During goals-of-care discussions (GoCDs) in hospitals, patients express their goals, fears, and worries, while health care providers share information about prognosis and treatment options [1]. The exchange of information allows clinicians and patients to create a plan of care that aligns with the patient’s wishes and is consistent with their goals [2]. Information sharing during GoCDs can play a critical role in preparing substitute decision-makers for future decision-making if a person is no longer capable [3]. Many patients lack documented GoCDs in their medical records, with recent studies showing documentation rates of only 21% to 36% [4,5].

These conversations do not occur due to several factors, including physicians’ fear that patients will have difficulty accepting poor prognoses, concern about conflict between family members, and physicians placing low priority on these conversations because of competing demands [4,6,7]. For patients, barriers include lack of knowledge, limited time during physician visits, and the expectation that physicians will raise the topic when necessary [8,9]. When GoCDs do not take place, patients report receiving treatments that are either more or less intensive than they would want and do not feel heard and understood by clinicians, resulting in increased anxiety and depression [10,11]. To address these barriers, we developed an interactive GoCD priming tool called MyCare with a team of patient partners, clinicians, researchers, and software designers. MyCare was created to (1) help patients who are seriously ill in hospitals express and communicate their fears, goals, and wishes for their health within their circle of care and (2) empower patients to initiate GoCDs with their family members and clinicians.

### Objectives

We aimed to conduct a pilot study of MyCare on internal medicine wards with patients who are seriously ill and physicians to assess its acceptability, usability, and feasibility. A secondary objective was to gather efficacy data to support a future randomized controlled trial.

## Methods

### Tool Development and Description

MyCare is an interactive web-based application designed to help patients express their goals, values, and wishes related to their health care. The tool was designed to (1) educate and empower patients to participate in their own care, addressing the patient barrier of lack of knowledge; (2) elicit information from patients about their informational needs, prognostic understanding, and need for support so that these needs can be communicated to their care team; and (3) give patients an opportunity to reflect, clarify, and express their goals as they cope with serious illness, targeting the barrier of perceived irrelevance of GoCDs [9,12]. Screenshots of the tool and a detailed description of the design, development, and testing of MyCare can be found in Multimedia Appendices 1 and 2. In brief, we first reviewed the literature on existing advance care planning tools. Next, we convened a group of key stakeholders, including palliative care and internal medicine clinicians, patients who have experienced serious illnesses, web designers, and graphic designers, to design MyCare. The key elements for MyCare were determined by input from stakeholders and the literature on existing tools [13,14]. Our initial draft was adapted and modified through 6 rounds of feedback with stakeholders. The final design was presented to the Equity-Mobilizing Partnerships in Community group to determine how MyCare meets the needs of diverse populations [15].

The final version of MyCare included questions about the understanding of the illness, informational needs, support networks, preferred language, level of independence before hospital admission, current symptoms, and a question from the dignity-preserving care movement [16,17]. MyCare also includes 3 fictional narratives of people living with serious illness who have different goals for their health. In one narrative, the person values living as long as possible more than comfort; in the second, the person values comfort more than living longer; and in the third, the person values living longer only if certain abilities are maintained and distress is limited. Users were asked to select the narrative where the person’s goals were most similar to their own. The final questions asked patients to select and rank their most important priorities, worries, and goals with both closed and open-ended questions. The final section presented a summary of the patient’s responses and instructions on how to share this information with family and physicians. Screenshots of the tool are provided in Multimedia Appendix 2.

### Study Design

We used a convergent parallel mixed methods design to evaluate the acceptability, usability, and feasibility of MyCare for inpatients and their attending physicians at an academic teaching hospital in Ottawa, Ontario, between May 2022 and November 2022 [18]. A convergent mixed methods design allows us to collect qualitative and quantitative data simultaneously, analyze them separately, and subsequently meld the results together to provide a more in-depth answer to our research question.

### Ethical Considerations

Our study was approved by the Ottawa Health Science Network Research Ethics Board (approval code OSHN-REB#20210787-01H), which approved all study material, including consent forms. All participants provided informed consent before participation and were not compensated. Study data were securely stored on hospital servers. Study data have been anonymized to protect participant identity.

### Participants and Recruitment

Study participants were recruited from the internal medicine wards at 2 tertiary care teaching hospitals in Ottawa, Ontario. Admitted patients were eligible if were comfortable communicating in English and met one or more of the following criteria: (1) aged ≥70 years; (2) 1-year risk of death >20% calculated by an automated, validated predictive model run within the electronic health record [19]; or (3) recommended for inclusion by their attending physician based on the potential benefit to the patient. Patients were excluded if they could not participate in a discussion about their care due to cognitive issues. If patients could explain the study elements and risks back to the research assistant during the consent process, they were deemed to have adequate cognition to participate. Consenting participants were asked if they would like to complete the study with a support person. After patient recruitment was complete, internal medicine physicians who worked at the 2 study hospitals during the study period were contacted via email and asked to participate in semistructured interviews.

### Intervention

MyCare was administered on an Apple iPad with the support of a caregiver or the research assistant. With consent, a copy of MyCare responses was sent to the attending physician in the hospital and uploaded into the electronic medical record.

### Data Collection, Outcomes, and Evaluation Framework

#### Fit Between Individuals, Tasks, and Technology Framework

We used the fit between individuals, tasks, and technology framework to guide our evaluation of the feasibility (the ability for a technology to be adopted into a setting), usability (how well a technology meets user needs), and acceptability (user-task fit) of MyCare [20,21]. The framework posits that adoption of a technology requires a fit between the user, the technology, and the task. We measured qualitative and quantitative outcomes and compared or combined the matching categories where appropriate. For example, the quantitative and qualitative usability findings were compared to provide a richer understanding of usability for our study population.

#### Outcomes and Data Collection

We measured usability (fit between user and technology) using 3 different methods. The eHealth literacy assessment tool measures user confidence with technology across 3 domains: using a computer, using a touchscreen, and finding information online [22]. We measured usability with the System Usability Scale (SUS) and semistructured interviews [23]. The SUS consists of 10 statements scored on a 5-point Likert scale and is normalized on a 100-point scale, with higher scores indicating greater usability. In addition, semistructured interview questions further explored the experience of using the MyCare interface (user-task fit).

We assessed acceptability through semistructured interviews with patients and physicians focusing on aspects of the tool they found useful, what could be improved and added to the tool, and what should or could be removed from the tool. Ethnographic field observations were collected during patient encounters to supplement data on acceptability.

We assessed feasibility by using MyCare with the target patients and physicians for whom it was designed, measuring the time required for patients to complete MyCare and semistructured interview questions and researcher memoing.

We measured advance care planning engagement as an exploratory efficacy outcome that could be used for a future randomized trial. The advance care planning engagement survey is a validated measure of behavior change in 4 advance care planning domains [24].

The semistructured interview guide is provided in Multimedia Appendix 3. Interviews were conducted in person and audio recorded by MK, and SR, both early-career, female research assistants with training in qualitative methods. Participants had no relationship with or knowledge of the interviewers before study commencement.

#### Analysis: Quantitative and Qualitative

We calculated Pearson correlation coefficients between the SUS and age, sex, and eHealth literacy scores. The advance care planning engagement survey was analyzed using two methods as suggested by the tool’s creators: (1) mean difference and (2) percentage of participants who were more ready to engage in advance care planning behaviors [25,26].

We used inductive qualitative thematic analysis to analyze our semistructured interviews [27]. One member of the research team transcribed interviews, field notes, and postinterview memos, while a second member reviewed transcripts for accuracy. Inductive thematic coding was used to depict themes from interview transcripts related to acceptability, usability, and feasibility of our tool for patients and physicians. Coding of transcripts was completed by 2 coders (MK and DK) in Microsoft Excel. DK is a midcareer, male, internal medicine physician scientist. One coder (MK) read multiple transcripts to establish a draft codebook, which was reviewed through iterative discussion with a second coder (DK) until consensus was met and a final codebook was created. We then tested the codebook with other transcripts to ensure no new codes were identified. The final codebook was uploaded to Excel to facilitate coding of transcripts. To ensure consistency, the first transcript was done via consensus coding (MK and DK), and then the remaining transcripts were coded (MK) alongside field notes and postinterview memos. Codes were organized into themes and subthemes according to acceptability, usability, and feasibility for patients and physicians. Both coders identified themes and subthemes and discussed them with a third team member (SR). Themes were then defined and described.

## Results

### Overview

There were 28 patients who consented, 25 (89%) completed the study, and 8 internal medicine physicians participated. In total, 3 (11%) patients withdrew due to declines in health, interruptions in medical procedures, fatigue, and discharge from the hospital before study completion. In total, 4 (16%) patients had a support person involved; it was up to the participant if they wanted to have a support person with them during the research encounter. Through memoing by the research team, we know that these encounters were comparable to those without a support person present. The mean age of participants was 76 (range 70-88) years, and 64% (16/25) were women. Most patient participants were White, English-speaking, Catholic, and lived at home before admission (Table 1).

**Table 1:** Characteristics of participants using the MyCare study tool (N=25).

Participant characteristics		Values
Age (y), mean (SD)		76.24 (6.46)
**Sex, n (%)**		
	Female	16 (64)
	Male	9 (36)
**Place of residence, n (%)**		
	At your home (apartment, townhouse, or bungalow)	20 (80)
	Hospital	1 (4)
	Retirement residence	3 (12)
	Other	1 (4)
**Ethnicity, n (%)**		
	East Indian	1 (4)
	Middle Eastern	1 (4)
	White	22 (88)
	Missing	1 (4)
**Primary language, n (%)**		
	English	21 (84)
	French	3 (12)
	Bilingual	1 (4)
**Religion, n (%)**		
	Catholic	12 (48)
	Atheist or agnostic	7 (28)
	Protestant	5 (20)
	Muslim	1 (4)
**Education level, n (%)**		
	Did not complete high school	1 (4)
	Completed high school	8 (32)
	Had university or postsecondary education (trade, technical, or vocational school)	8 (32)
	University degree	5 (20)
	Graduate degree	3 (12)
**Annual household income (CAD $^a^), n (%)**		
	<10,000	1 (4)
	10,000-19,999	1 (4)
	20,000-34,999	3 (12)
	35,000-49,999	3 (12)
	50,000-74,999	4 (16)
	75,000-99,999	6 (24)
	>100,000	2 (8)
	Missing	5 (20)

^a^CAD $1 was US $1.30 at the time of the study.

Patients took a median time of 32 (IQR 21-43) minutes to complete MyCare, with a range of 12 to 101 minutes. The mean eHealth literacy score was 56 out of 100. In total, 19 (76%) of the 25 patients indicated that MyCare helped them think about and understand what is most important to them, and 16 (64%) stated MyCare helped them think about the type of care they would want if their illness progressed.

The mean advance care planning engagement scores on a 5-point scale before and after MyCare were 4.11 (SD 0.77) and 4.20 (SD 0.74), respectively (mean difference 0.09, 95% CI –0.08 to 0.34; *P*=.22). After completing MyCare, 16% (4/25) of the patients reported feeling more prepared to talk to their medical decision-maker about the type of care they would want if they were very sick or near the end of life, and none felt less prepared. Moreover, 12% (3/25) of the patients felt more ready to ask their physician questions, while 4% (1/25) felt less ready. In addition, 12% (4/25) of the patients felt more ready and 12% (4/25) felt less ready to talk to their physician about the kind of medical care they would want if they were very sick or near the end of life.

### Usability

The mean SUS score was 64.7 out of 100 (range 42.5-80.0) or “okay” using the adjective rating scale presented by Bangor et al [28]. The mean score for internet-based webpages and applications is 68.1 [23]. Patients’ technology confidence was positively correlated with SUS scores (*r=*0.49; *P*<.05). Age and sex had no correlation with the SUS score. Consistent with the “okay” score, some patients required assistance from research staff to navigate MyCare, read questions, and select answers.

### Semistructured Interviews

Interviews with patients and physicians uncovered themes related to usability, feasibility, and acceptability. Thematic codes, definitions, and exemplary quotes from patient and health care provider interviews are provided in Multimedia Appendices 4 and 5.

#### Usability

Within the usability theme, we found facilitators and barriers. Patients were motivated to use the tool because it was easy and enjoyable and empowered them to initiate and participate in conversations about their care. Barriers for patients included needing physical and technological support to use the iPad and navigate the MyCare interface, repetitive questions, and difficulty knowing how to answer some questions. Patients commented that prespecified response options for questions did not always capture the complexity of their responses or allow for flexibility in responses that may change depending on their state of health. Patients asked for free text fields to express themselves:

A lot of the questions are yes or no answers but a lot of them are not yes or no answers, they are in between. Or there is an explanation to the yes and the no and that is not provided. I think it should say yes and qualify, or no and qualify.

Being able to provide context or additional information that is not captured within a yes or no answer was important. Some patients commented that they had difficulty using technology, including touch screens.

Physicians thought that having the patient’s wishes documented in the MyCare output would help them keep family meetings focused on the patient’s values. A physician shared the following:

Goals always get made. Just it’s the confidence of which you’ve made them right. Whether you’ve talked to enough family? Or whether or not it’s the first time you talk to Mr. Smith? Was he truly with it or not so much. Right? And then the family come and they doubt your word.... So having extra information is always very helpful.

Physicians expressed that sharing patients’ responses with the health care team would promote shared decision-making, as the MyCare results are uploaded to a patient’s electronic medical record. Physicians thought that MyCare could serve as a framework for junior staff or trainees who have less experience facilitating GoCDs. In addition to serving as a framework, the tool could help patients and physicians reach a common understanding. Discussing questions and responses from MyCare could help physicians initiate GoCDs around specific topics that may be difficult to introduce.

#### Acceptability

Patients and physicians provided insights about the perceived utility of the tool and made suggestions for improvement, an aspect of user-task fitness and acceptability (Multimedia Appendix 6). Patients said the most useful aspects of the tool were questions about goals after hospital discharge, the type of care they would want near the end of life, and the people who are most important to them. Patients thought that questions were repetitive, which they found frustrating. Patients suggested adding questions about mental health, specific preferences for end-of-life care, and community care would better reflect the needs of the target patient population. Patients self-identified 2 functions of MyCare: personal reflection and supporting shared communication of their goals, wishes, and values. The patient stated that MyCare was informative and allowed them to think about next steps as they age:

[T]he part about getting older and close to death and prioritizing and reflecting on the things, that made me aware to do the things.

Patients appreciated that the tool helped them articulate what is most important to them and think about future decisions they may face. Furthermore, patients planned to keep their responses for their own reflection and to ensure their wishes were respected.

Some patients felt they already knew what they wanted and did not need the tool to guide them; these patients still planned to use the tool to initiate and guide communication with their families and health care team. One patient said that although the tool would not be useful to them, it “might be [helpful] to the family to show it to the kids and give them an understanding of how I feel without having to say.”

Another patient shared that by using the tool “the doctor can clarify what lies ahead for [me] that I don’t realize right now...and to help me understand why I’m going through certain steps.”

For some patients, completing MyCare reinforced that they had been talking to their physicians about the right things, which increased their confidence in their health care team and their own ability to engage in conversations about their care.

However, the value of MyCare was limited for some patients who shared that the tool content did not relate to their experiences or that they would prefer to talk to their physician about their goals of care instead of using an online tool. Several patients voiced concerns that the tool would only be useful if “the doctors are able and willing to listen and would take action to what I’m saying.”

Some patients doubted the tool could improve their health care experiences related to GoCDs.

Physicians reported that the most useful aspects were asking patients to prioritize their most important goals and to share their illness understanding, while the least useful aspect was the symptom scale because this information is already obtained during routine clinical assessment. Physicians suggested more ranking tasks could be integrated for goals, wishes, fears, and symptoms to identify what is the most important to the patient. There was mixed feedback about the description of patients’ activities of daily living. Some physicians found it helpful to know what activities of daily living patients could complete before coming to the hospital, while others indicated it was less important than knowing what they want to be able to do when they leave.

Physicians said they would trust and value the patient’s responses to MyCare questions if they knew responses were up-to-date and received the results promptly. One physician shared that they would be motivated to use the tool with their patients if they knew it was supported at an institutional level by the hospital. Others suggested that integration of MyCare into the hospital workflows and explicit links to legal frameworks for decision-making would allow physicians to feel confident and supported in abiding by their patients’ wishes documented through MyCare.

Physicians found MyCare valuable to support reflection and shared decision-making. Physicians thought MyCare would help patients reflect on what was most important to them and help physicians to get to know the patient before meeting with them. By reviewing patients’ responses ahead of meeting with them, physicians suggested that the tool could promote shared decision-making between patients and their health care team:

I think what [the tool] does is it really sets the stage for understanding what [the patient’s] values are. So I definitely think you know, getting it before I had a goals of care discussion with the patient would be really helpful. I think for me to understand where that coming from, but also even for them to start getting them thinking about these things.

Physicians shared that MyCare results would improve their confidence that decisions align with the patient’s values, especially in cases where patients later lost the ability to participate in decision-making and the family’s wishes do not align with the patient’s.

Emotional responses to MyCare, captured in researcher memoing, were common. Some patients seemed empowered and confident in using the tool, making comments about how the questions were great and improved their confidence in telling others what is most important to them. Other patients expressed feelings of fatigue due to the length of the tool. Researchers’ memoing highlights that patients struggled with how long it took to complete MyCare in addition to studying questionnaires. Some patients expressed confusion and worry because the tool talked about living with serious illnesses, which they did not consider themselves to have or had not given much prior thought. Conversely, some patients did not seem to express positive or negative emotions while using MyCare.

#### Feasibility

Physicians were concerned that symptomatic illness would be a barrier for some patients, preventing them from engaging with MyCare. The concern was validated by memoing of fatigue for patients completing MyCare. In addition, physicians shared mixed perspectives on how time constraints would impact their ability to review MyCare results. Some felt MyCare would help facilitate GoCD’s under time constraints, stating the following:

[P]art of the challenge while on service is not enough time to do the necessary background, you know, getting to know the patient. So this helps you get a step ahead and gives you the information available to then have the conversations.

While others indicated that they would not have time to review the information from MyCare after the patient completed it.

## Discussion

### Principal Findings

We designed and pilot-tested an interactive online GoCD priming tool with patients who were seriously ill and admitted to the hospital’s general internal medicine ward. The piloted version of MyCare had a below-average SUS score [28]. Length of the tool and familiarity with touch screen technology were the primary usability issues and could be improved by removing the symptom assessment and activities of daily living questions that patients and physicians thought were unnecessary. MyCare had moderate to poor user-technology fit, but we think this can be improved by pushing further toward minimalist design with succinct content as suggested by the study participants and guidelines on user interface design for older patients [29,30]. Patient concerns with inflexible response options could also be improved with optional free text fields to allow for in-depth answers while maintaining prespecified options.

Overall physicians found the MyCare results highly usable, allowing them to quickly absorb key information about patients’ goals and serve as a guide for the patient, physician, and junior trainees learning to have GoCDs.

MyCare had moderate acceptability. Most acceptability data from patients was related to perceived utility, the fit between task and individual. Many advance care planning interventions have poor acceptability because patients perceive them as irrelevant because they do not see themselves as having a high risk of death or decline even when they have a high risk of death [31]. We attempted to address this barrier by testing MyCare with people who are hospitalized for acute illness and have a high risk of death. Some patients commented that this context made GoCD more relevant, motivating them to use MyCare and share results with loved ones. MyCare also used narratives to demonstrate how real people have benefited from GoCD. Despite these attempts to target the barrier of “perceived irrelevance,” we were only partially successful. GoCD and advance care planning are complex behaviors with multiple barriers. Any future implementation of MyCare needs to target these multiple barriers using implementation science approaches to maximize uptake [32,33].

Future versions of MyCare and related tools can improve the task-individual fit by ensuring that tools meet the needs of patients and physicians. Patients told us that identifying goals after discharge, care preferences for the end of life, and the people who support them were the most helpful parts of MyCare. Physicians focused on prioritizing patients’ values, wishes, and goals for care to support medical decision-making. Although patients and physicians had different priorities, both agreed that values and goals for care were important and useful. Future versions of MyCare and similar tools should focus on shared patient-physician priorities, such as eliciting value-based information about what patients want. Values-based information is a shared priority and is more useful to substitute decision-makers than treatment-based information that only applies to a single decision [34]. Patients and physicians in our study said that MyCare helped to clarify values and could improve communication about these values between substitute decision-makers and medical providers.

We found several feasibility challenges. Patients took considerable time to complete the tool, required assistance, and became fatigued. Physicians thought that MyCare results would improve their care by allowing them to quickly understand what is most important to their patient, but the physicians we interviewed had not all received MyCare results when they were on busy clinical service. We do not know if MyCare will truly address the barrier of “not enough time” commonly reported by physicians [8]. Implementation of MyCare will require institution-level support to integrate MyCare into workflows, electronic medical records, and institutional documentation standards [35]. Stripping MyCare and the response summary to essential elements will further improve usability and feasibility by minimizing time investment for physicians and patients.

Given that GoCDs are not prioritized by physicians, tools must empower patients to initiate conversations or prompt physicians to do so. Patients need to know that information about their goals and wishes will have direct benefits for them, and they need tangible steps they can take to initiate these conversations. Patients can be further empowered by being given explicit control over who sees their responses. Finally, MyCare will be more effective if it is introduced by the patient’s physician, who commits to discussing the results. If patients share sensitive information using MyCare only for it to be disregarded by their team, it could break patient-physician trust and discourage future GoCDs.

### Strengths and Limitations

Our study has several strengths. Our study is grounded and organized around the fit between individuals, tasks, and the technology framework. Our convergent parallel mixed method design allowed us to collect and analyze both objective and subjective data around the use of MyCare, increasing reliability and depth. Another strength is that we implemented our tool in a real-world setting, an academic hospital with patients who are seriously ill and who benefit from GoCDs.

Our study had several limitations. First, our sample was younger, healthier, and less diverse than the tool’s intended target population. Some age-eligible patients were unable to consent or complete the study because of symptoms such as fatigue and cognitive impairment. For those who did complete MyCare, data collection was time-consuming. Participant fatigue contributed to missing data from the most unwell patients and may have biased results. Limited sociodemographic diversity may reduce generalizability, and therefore, testing with diverse populations will be important as we move forward. The highlights of this study are provided in Textbox 1.

**Highlights of the study.**

- We co-designed an online survey tool to help patients who are frail reflect on and share their goals, wishes, and fears with their care team and people important to them.
- Patients and physicians found the tool helpful to clarify patient goals, identify people who support the patient, and facilitate conversations.
- Our tool would be more usable if it were shorter and focused only on shared physician-patient priorities.

### Conclusions

Patients and physicians found the MyCare tool valuable for promoting conversations about goals of care. To improve usability and utility, MyCare and future tools must empower patients, prioritize simplicity, and limit content to areas that meet the shared patient’s and physician’s priorities.

## Appendix

Multimedia Appendix 1 MyCare tool development.

Multimedia Appendix 2 Screenshots from the MyCare tool.

Multimedia Appendix 3 Semistructured interview guide.

Multimedia Appendix 4 Patient interview themes.

Multimedia Appendix 5 Physician interview themes.

Multimedia Appendix 6 Suggestions for improvements.

## Abbreviations

GoCD: goals-of-care discussion
SUS: System Usability Scale

## Data Availability

Data are available upon reasonable request to the corresponding author.
