# Supplementary material for: Evaluation of a Novel Goals-of-Care Discussion Priming Tool (MyCare) in Inpatient General Internal Medicine Ward Settings: Feasibility, Acceptability, and Usability Study
Source: JMIR Form Res. 2025 Oct 28;9:e66932. doi: 10.2196/66932 (PMC12605267; doi:10.2196/66932)
Supplement: Multimedia Appendix 1 [file formative_v9i1e66932_app1.docx]

**Appendix 1:** MyCare Tool Development

The MyCare tool is an interactive online web application designed to help patients express their goals, values, and wishes related to their healthcare. Our development team included a varied group of key stakeholders and experts including palliative care and internal medicine clinicians, patients who have experienced serious illness, web designers, and graphic designers. Our development process started with team meetings to discuss the purpose and intent of the tool. The tool was designed to perform three functions: 1) to educate and empower patients to participate in their own care; 2) to elicit information from patients about their informational needs, prognostic understanding, and need for support so their care team knows how to engage them; and 3) to give patients an opportunity to clarify and express their goals as they cope with serious illness. A draft of the tool was created based on the intended purpose and then refined through four rounds of feedback within our development team.

To understand if our tool meets the needs of diverse populations, we partnered with the Equity-Mobilizing Partnerships in Community (EMPaCT) group^1^ for an additional feedback. The EMPaCT committee consists of patients and people with a range of diverse and intersectional lived experiences. This includes diversity related to: Language and Communication Skills, Housing, Education, Age, Ethnicity, Culture, Income Security, Sexual Orientation, Immigration status, Religion and Belief, among others, with the aim of centering diverse lived-experiences within projects to increase inclusivity and health equity in research. ^1^ We submitted a request for consultation to discuss the MyCare tool and shared the tool with them. We subsequently received a written report from the EMPaCT team with written recommendations that all members of EMPaCT have validated, ensuring that diversity and intersectionality have been addressed properly. Modifications to MyCare were completed based on their recommendations.

Privacy and control of personal health information were major concerns brought up during our consultation with EMPaCT, especially for groups with historical distrust in healthcare or prior negative healthcare experiences.

In the MyCare tool, patients are asked to answer questions related to what their hobbies are, their understating of their illness, tasks they would like to be able to complete post hospitalization. MyCare has a section with three different patient stories where the user can identify which of the three stories, they most identify with in terms of healthcare decisions. This indicates whether they most likely wish to have all possible lifesaving treatment even if there are risks, whether they wish only pain or symptom management, or prefer somewhere in the middle. The stories list out the hypothetical patient’s health problems, desires, and wished for their care. For example, one woman wants to get back home and continue with her favourite hobby of gardening and is willing to try treatment that will accomplish this goal.
