# Supplementary figures and images for: Evaluation of a Novel Goals-of-Care Discussion Priming Tool (MyCare) in Inpatient General Internal Medicine Ward Settings: Feasibility, Acceptability, and Usability Study
Source: JMIR Form Res. 2025 Oct 28;9:e66932. doi: 10.2196/66932 (PMC12605267; doi:10.2196/66932)

**Appendix 2:** Screen Shots from MyCare Tool


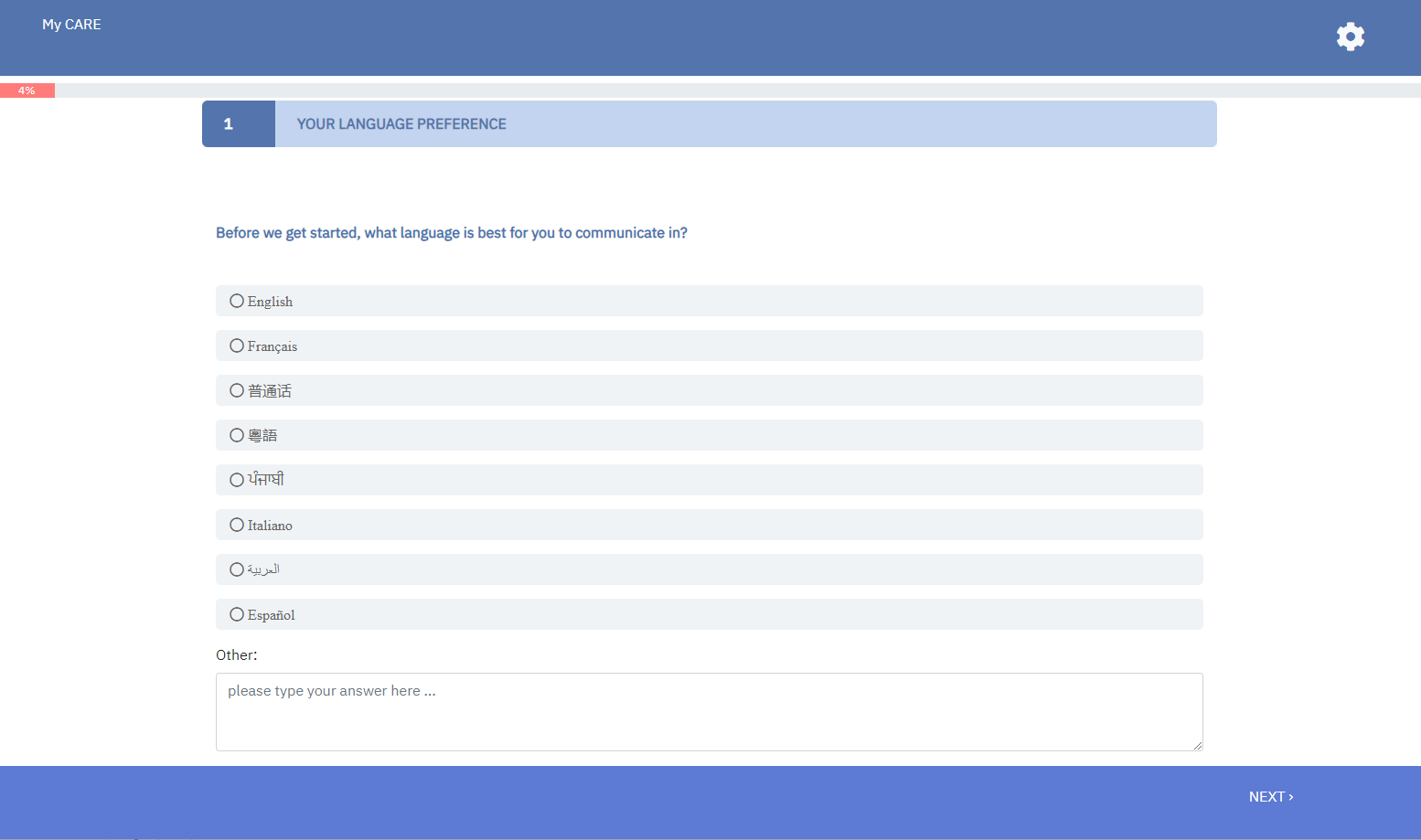


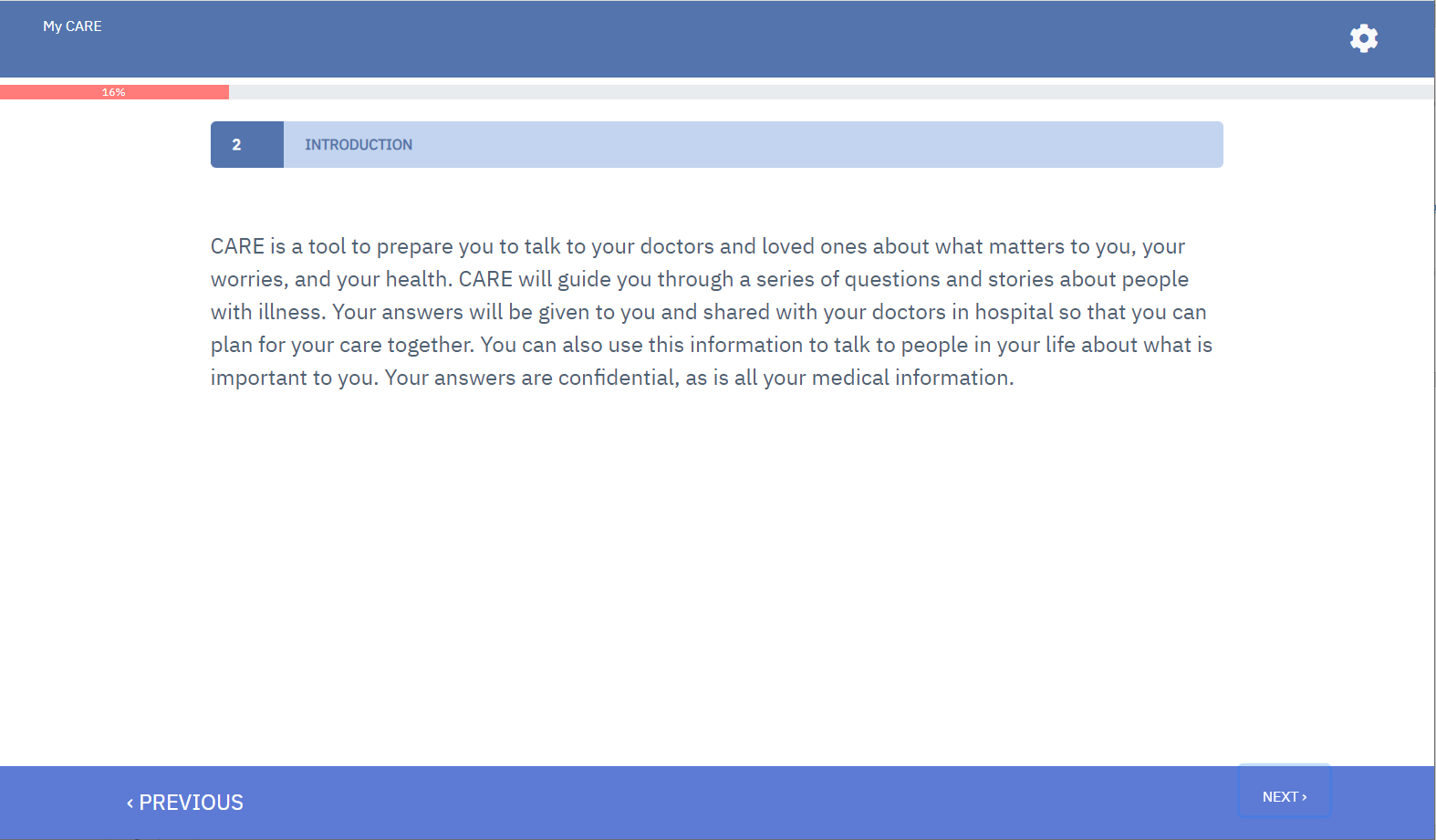


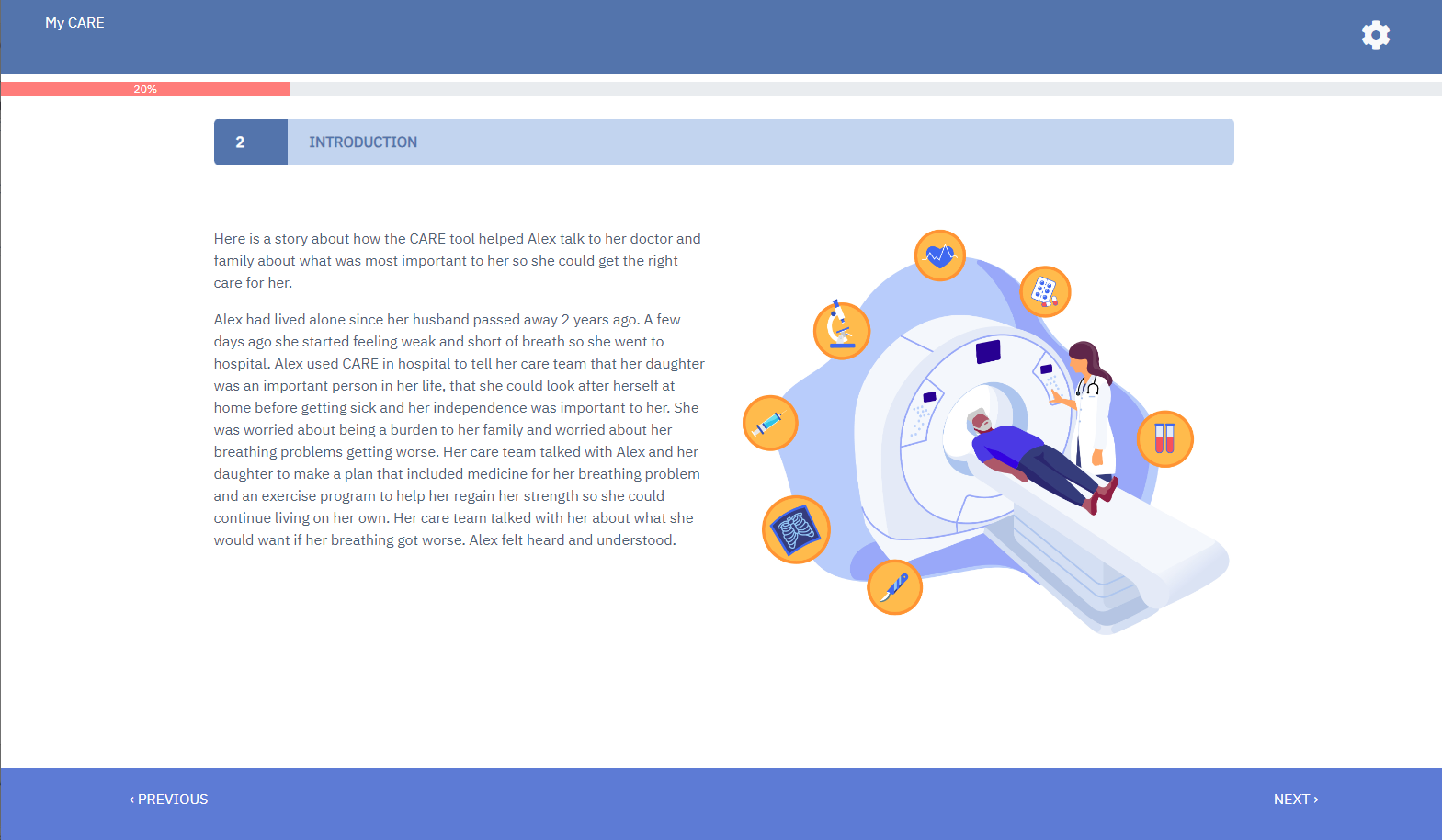


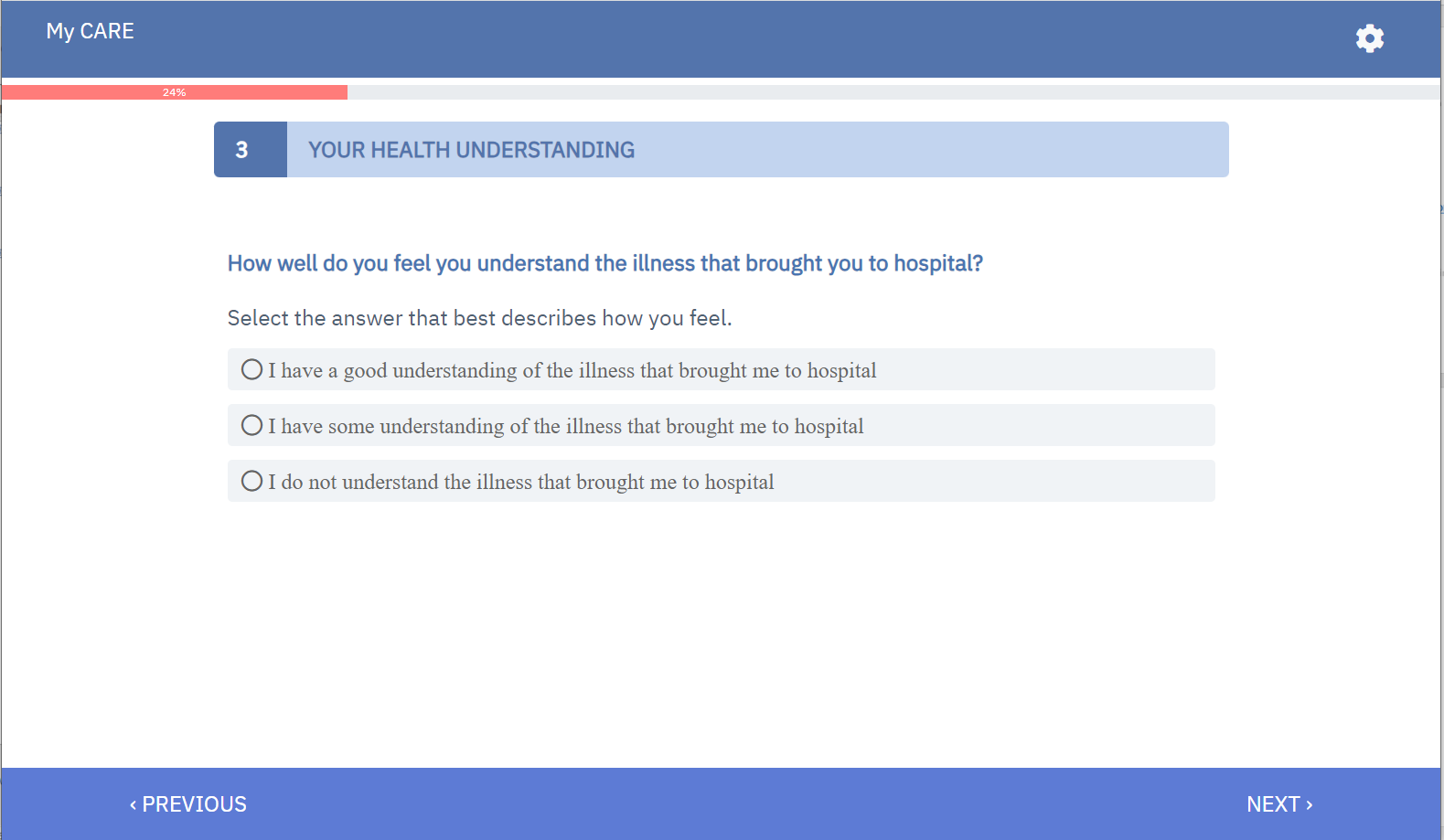


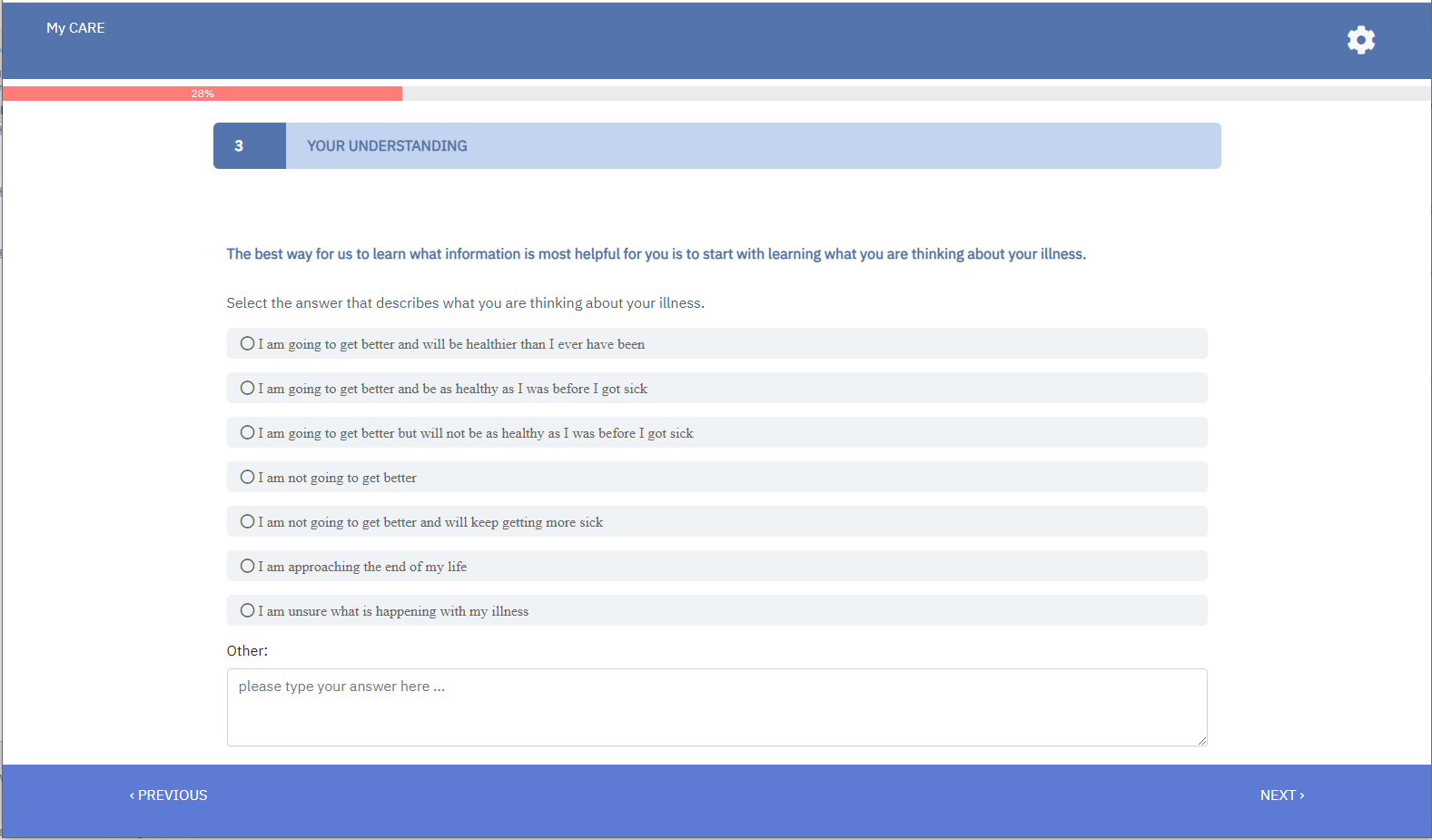


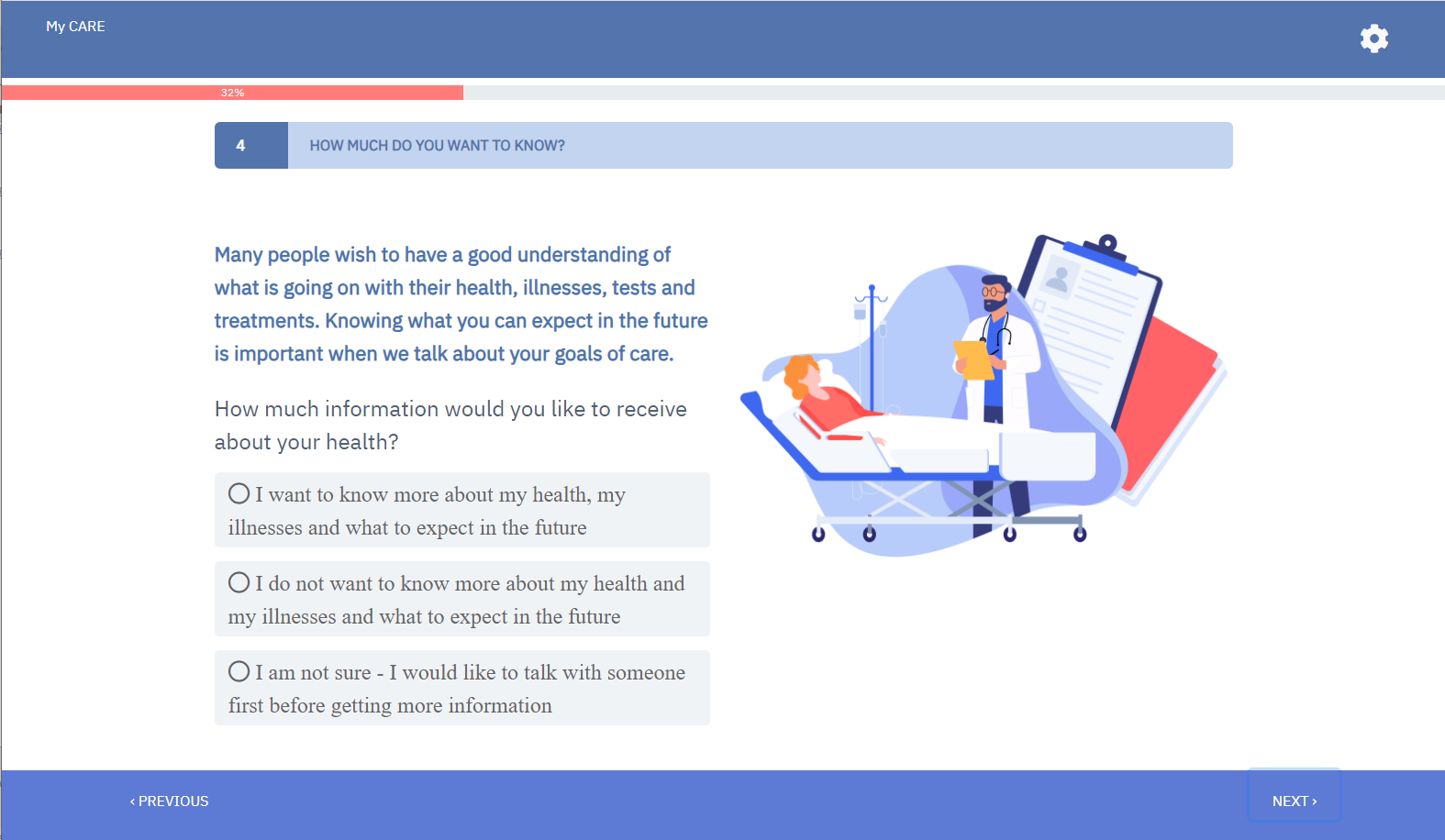


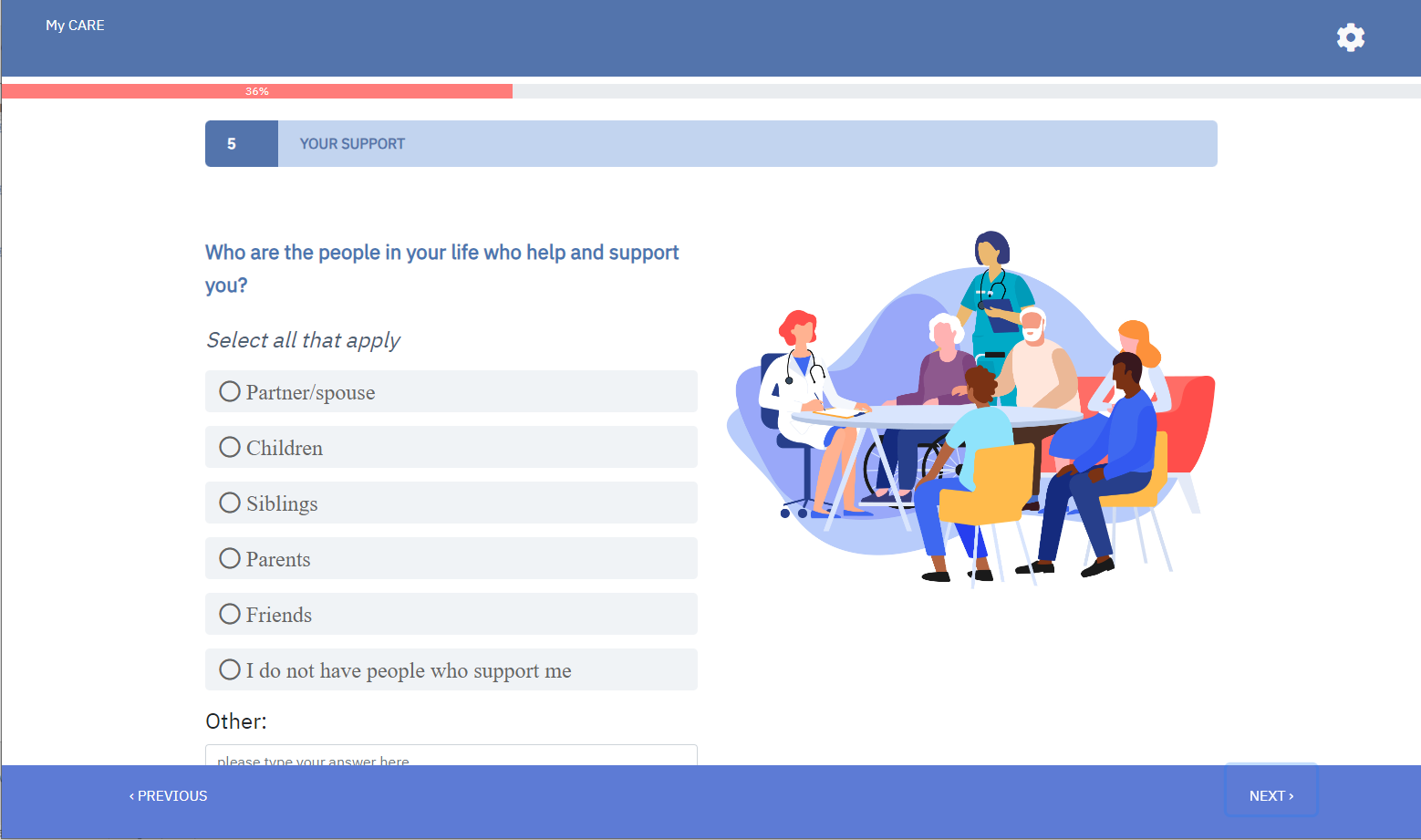


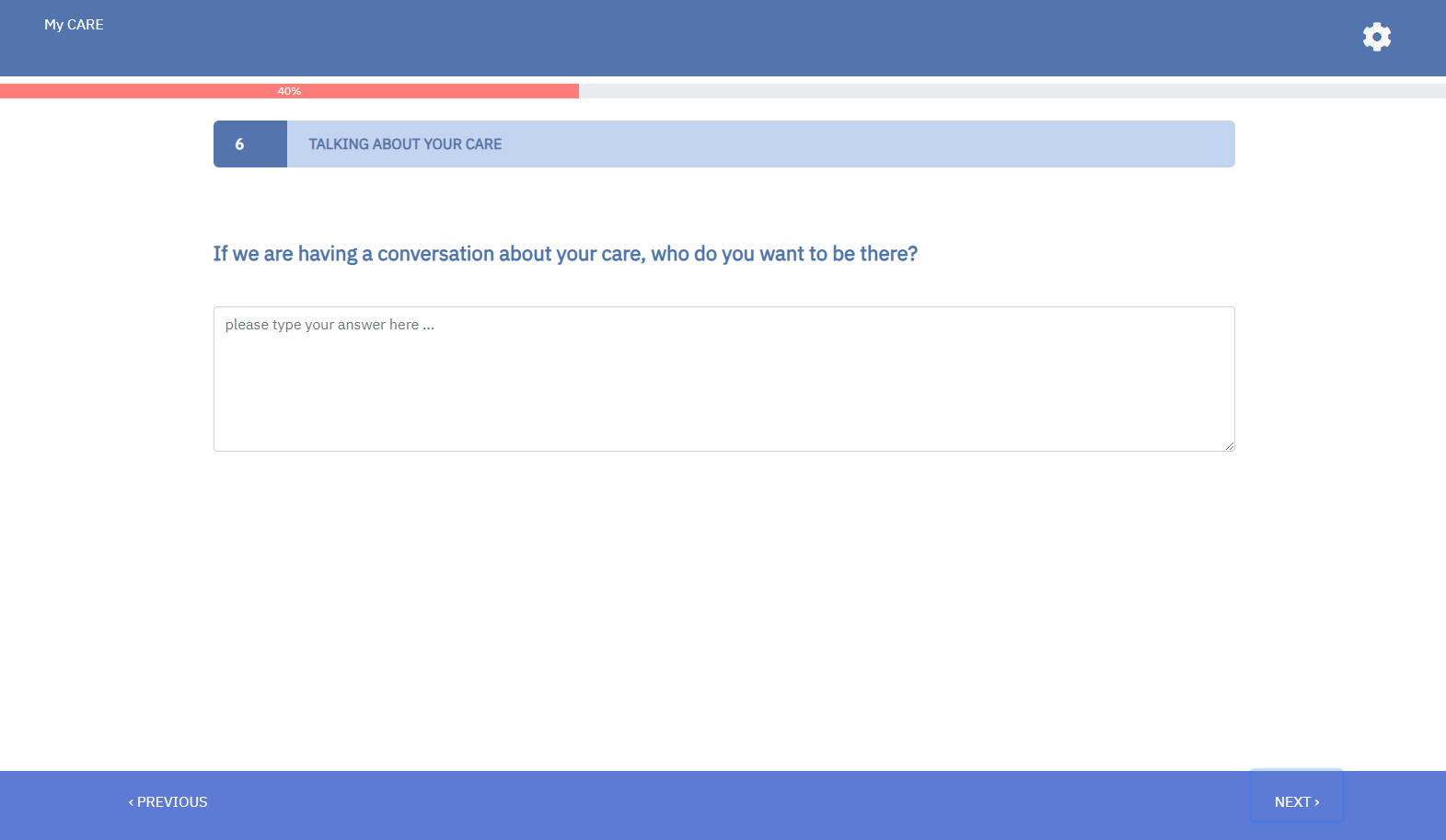


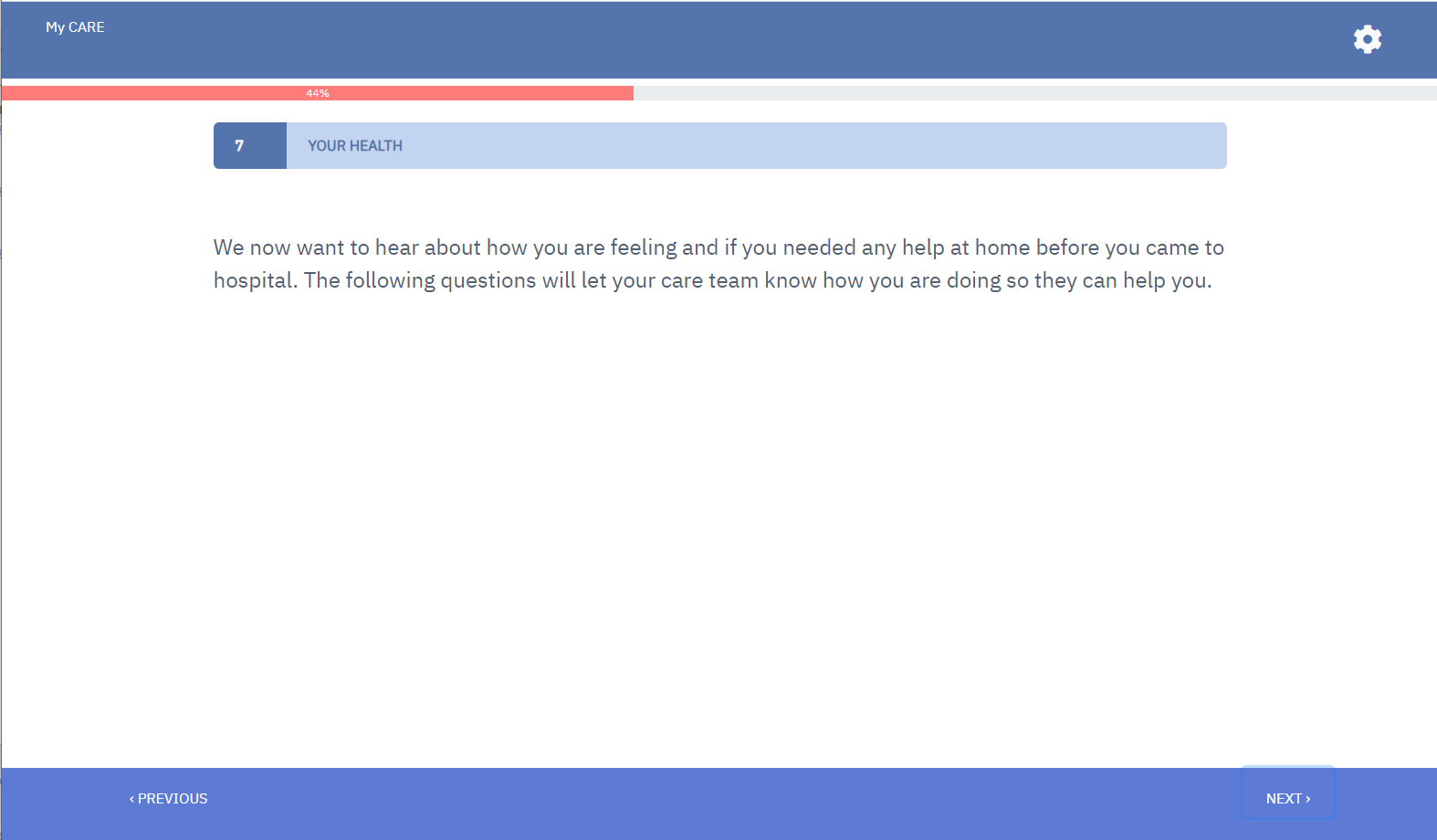


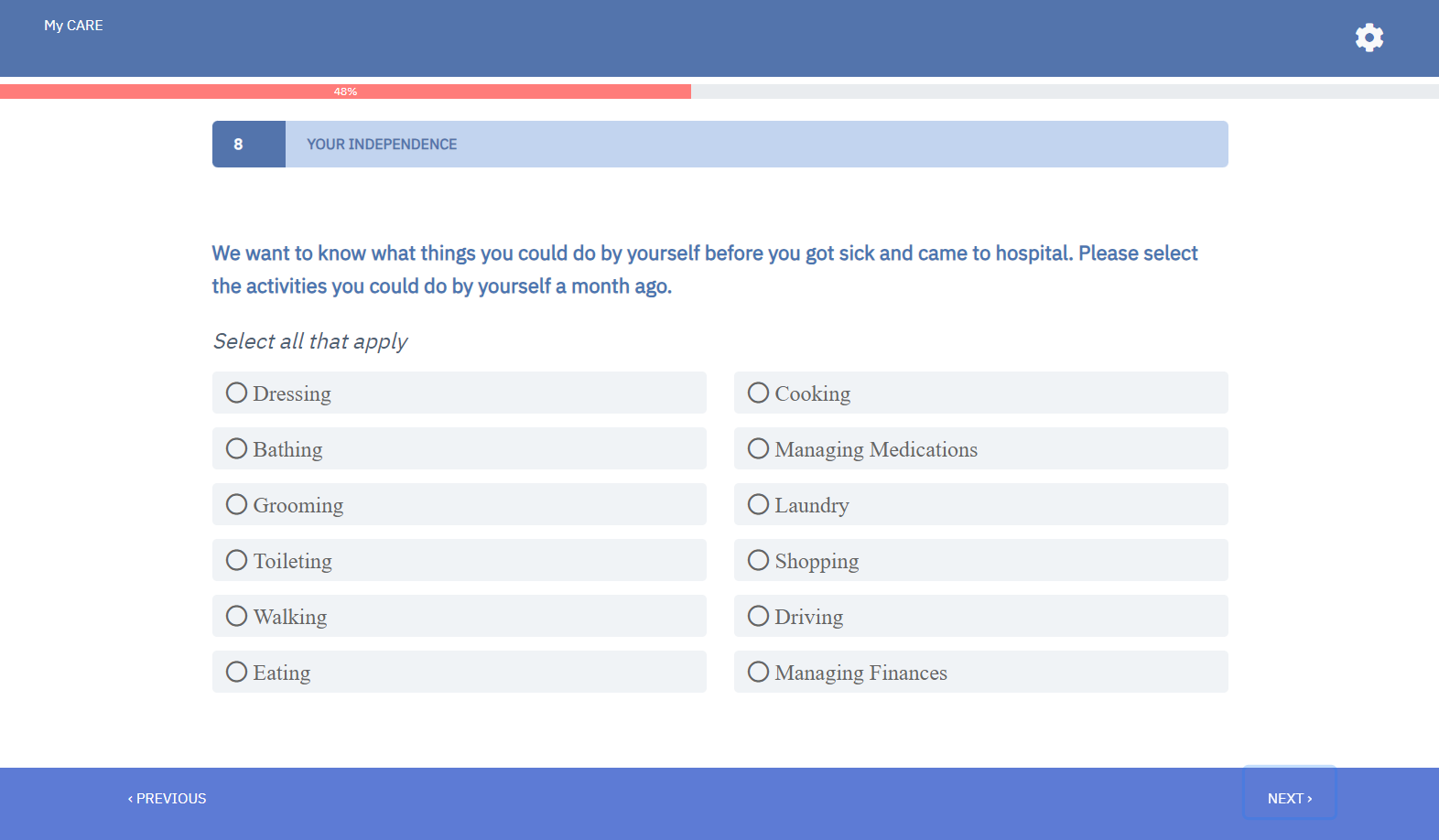


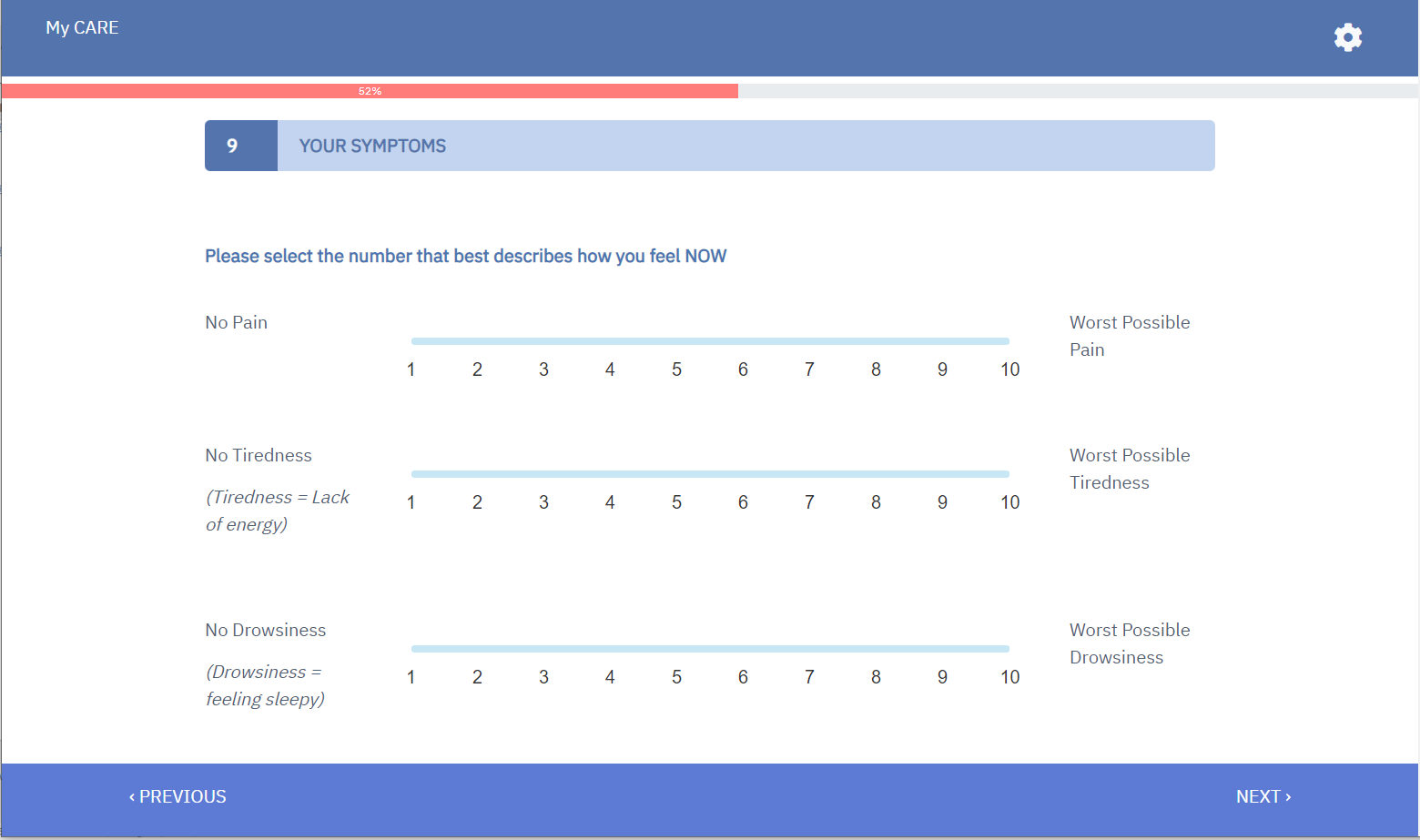


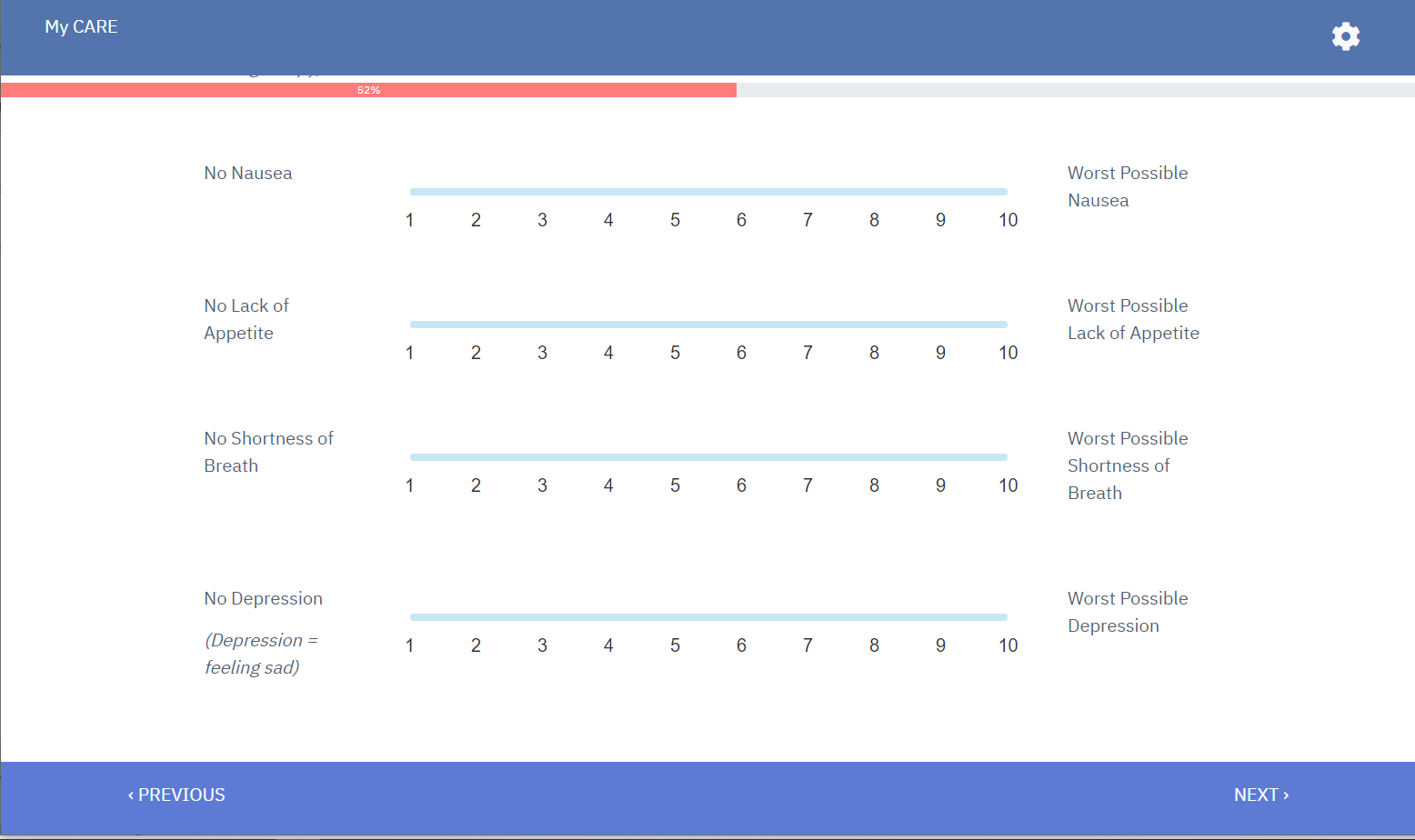


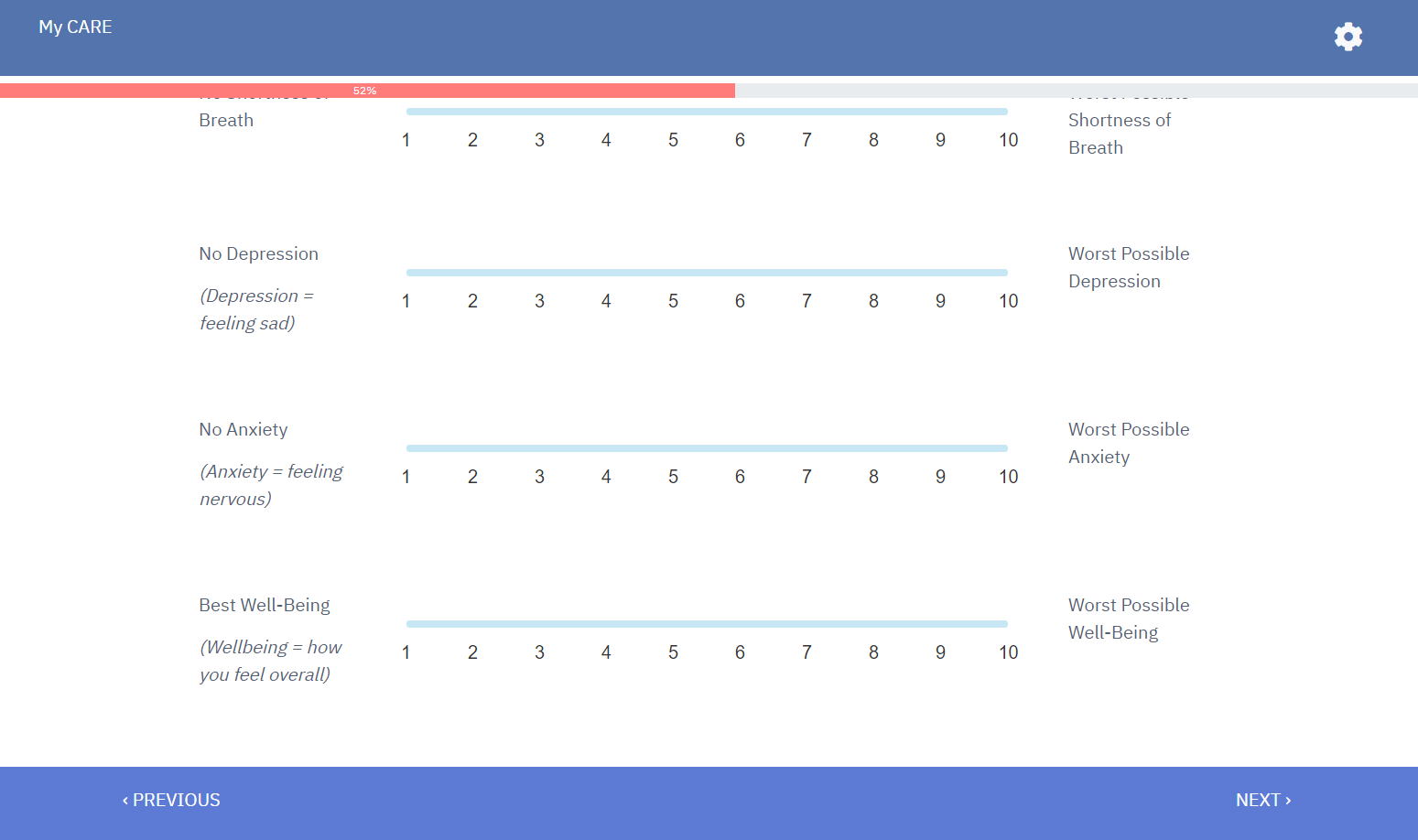


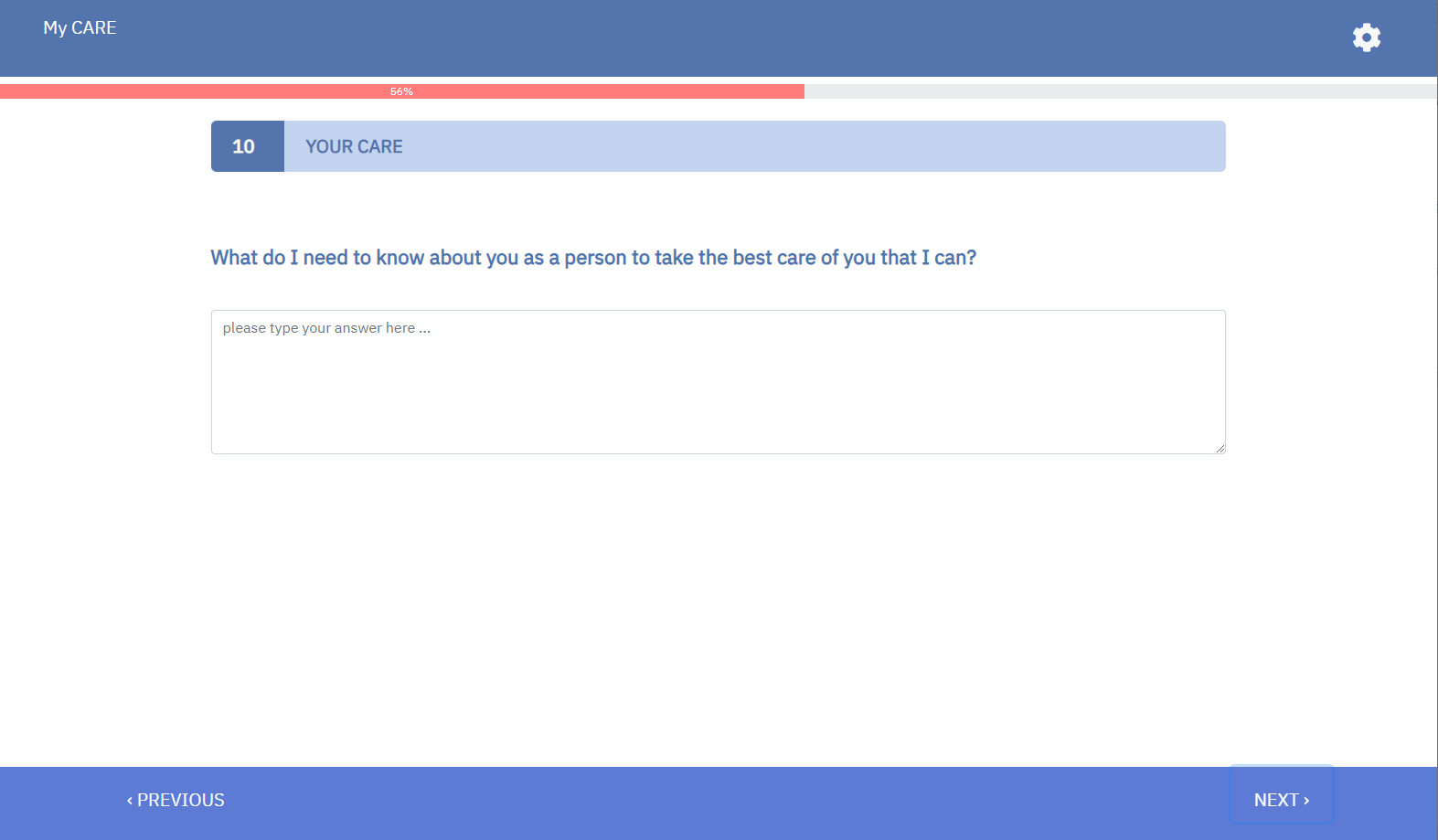


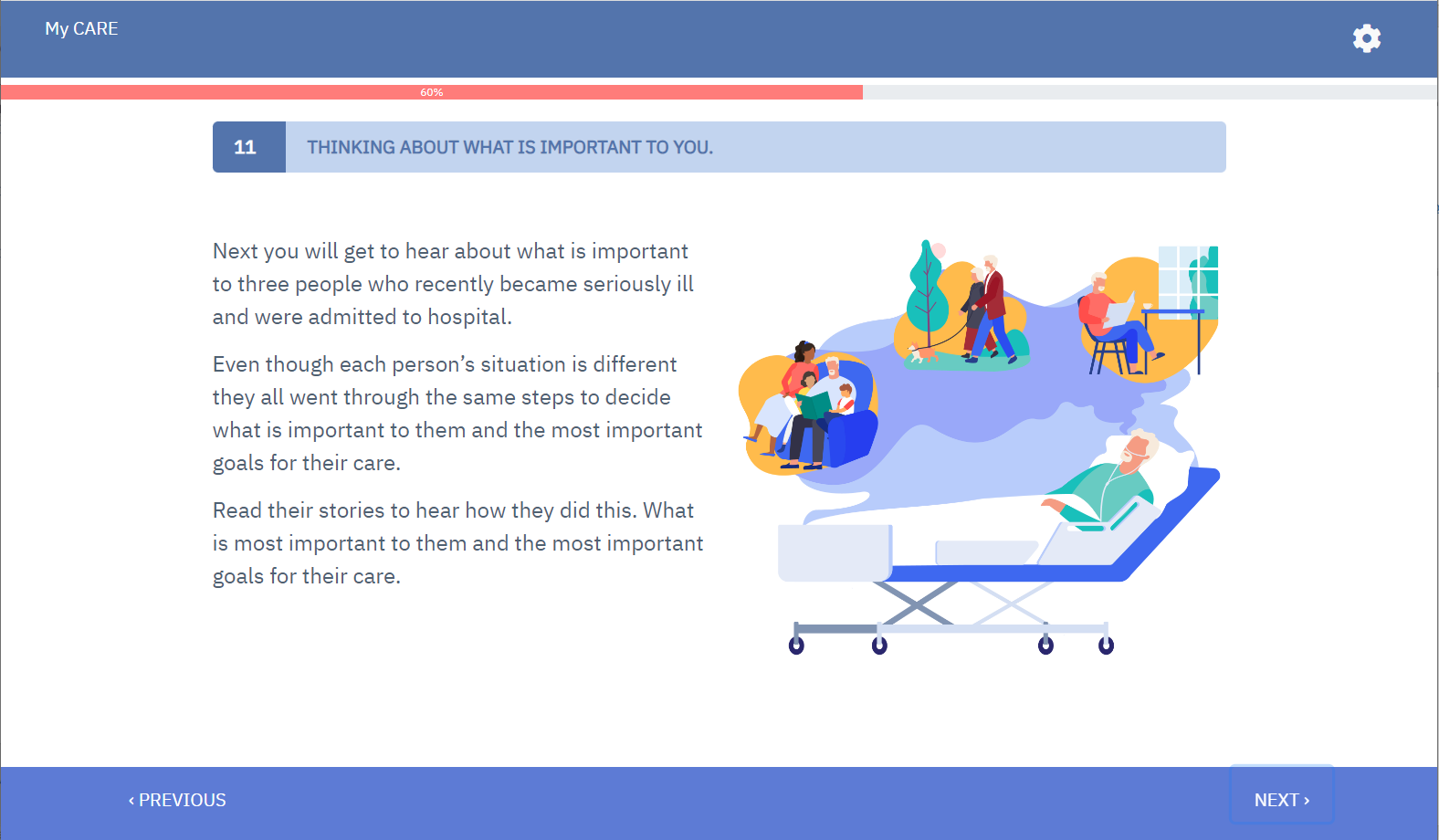


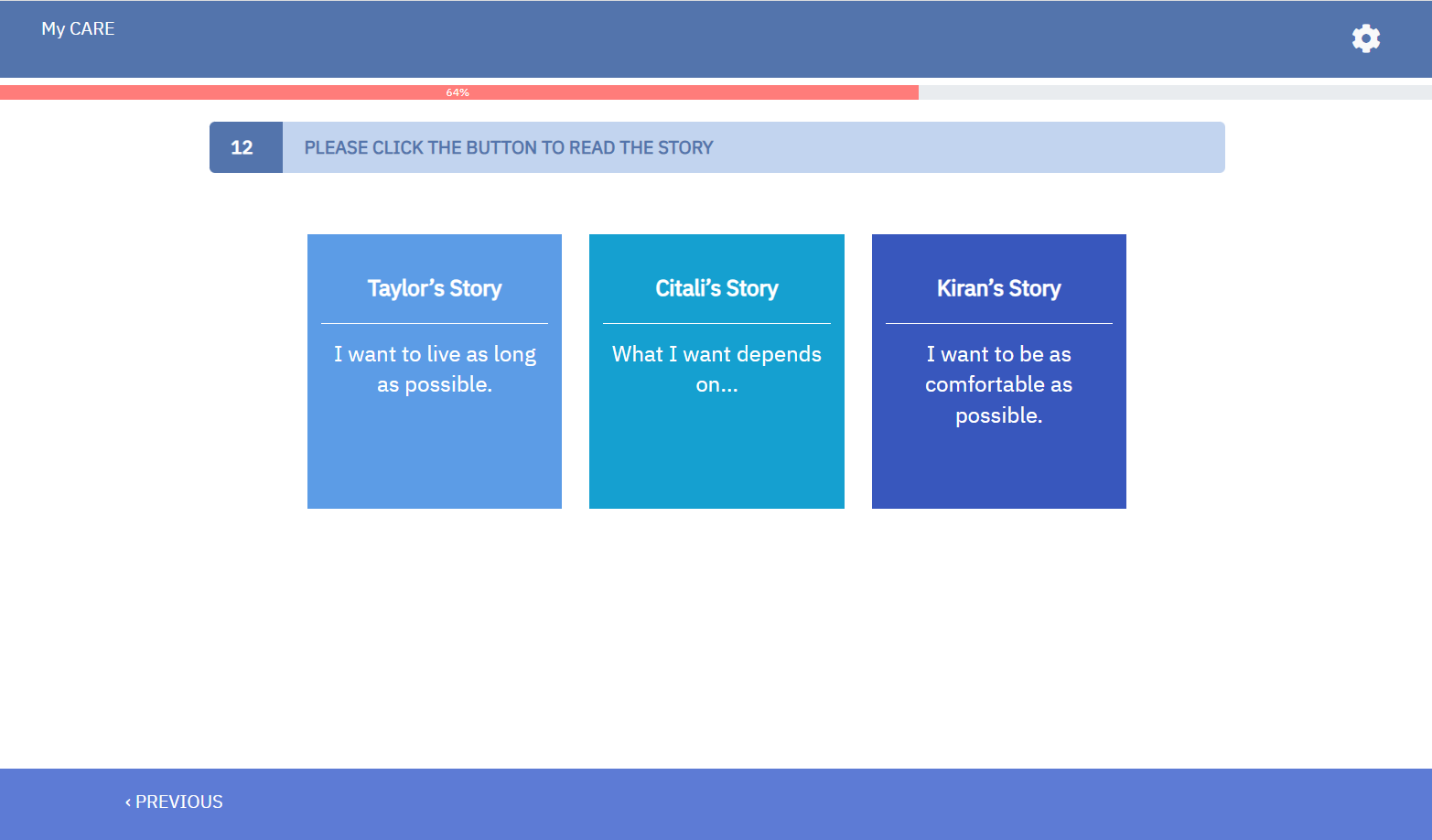


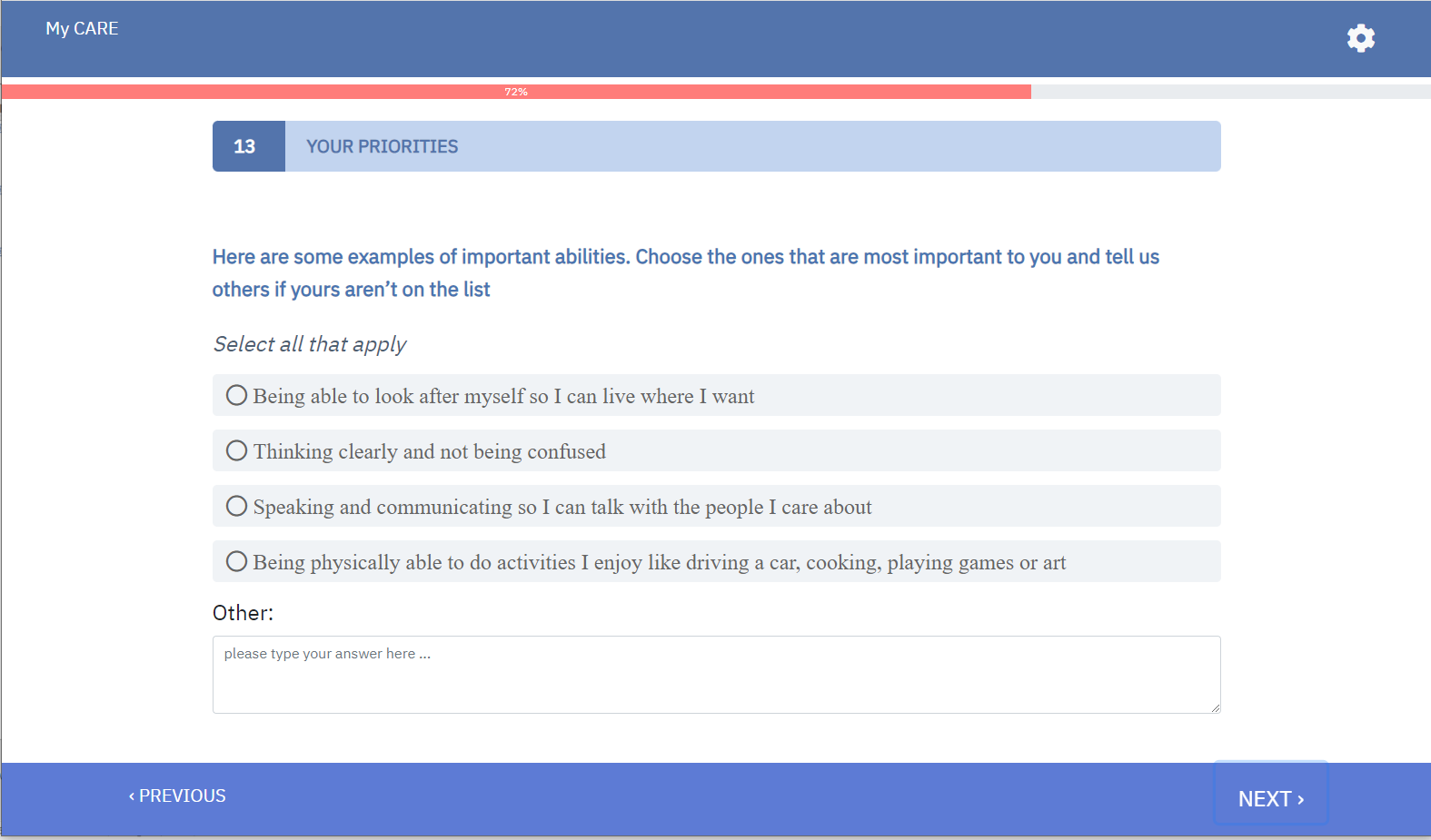


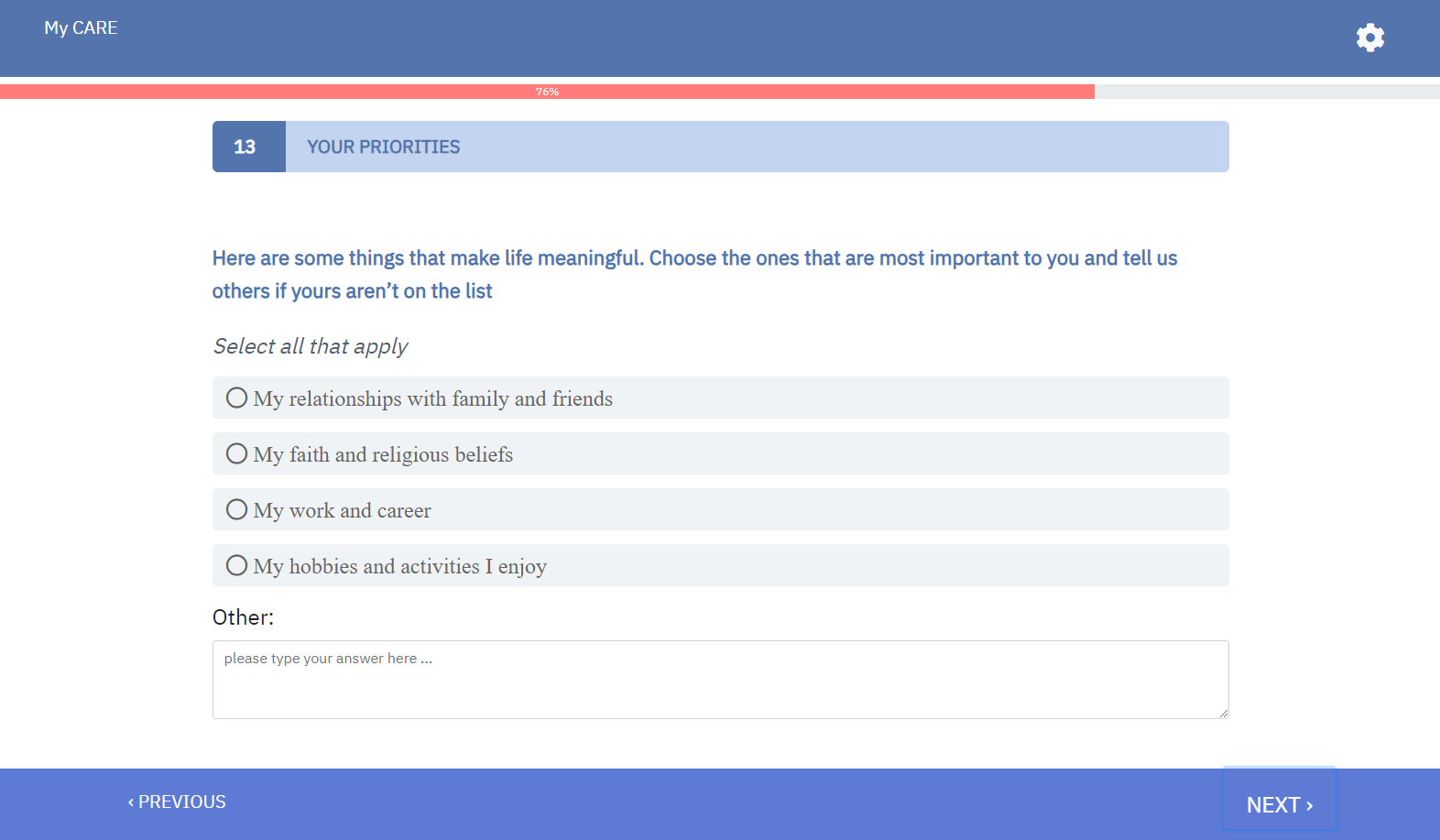


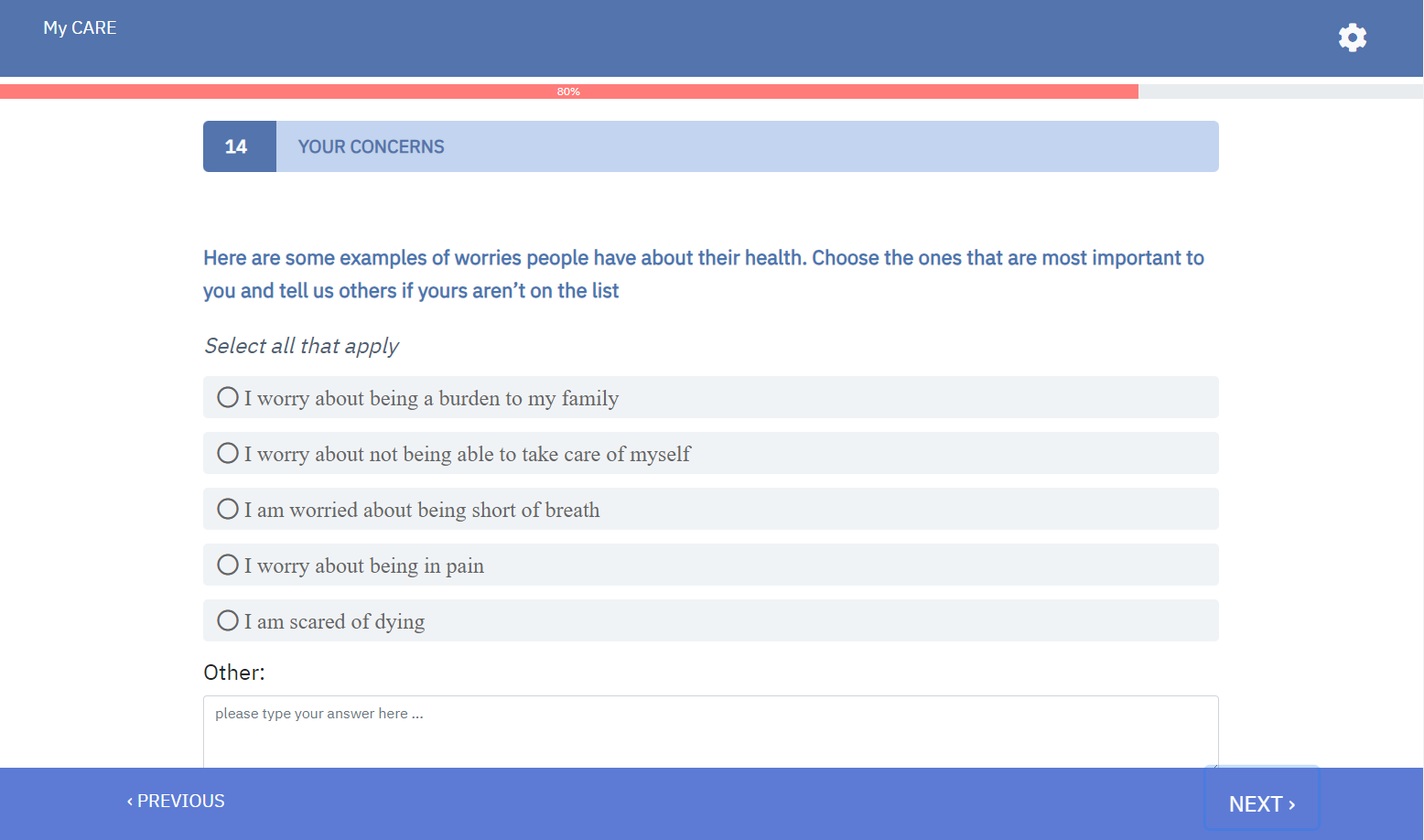


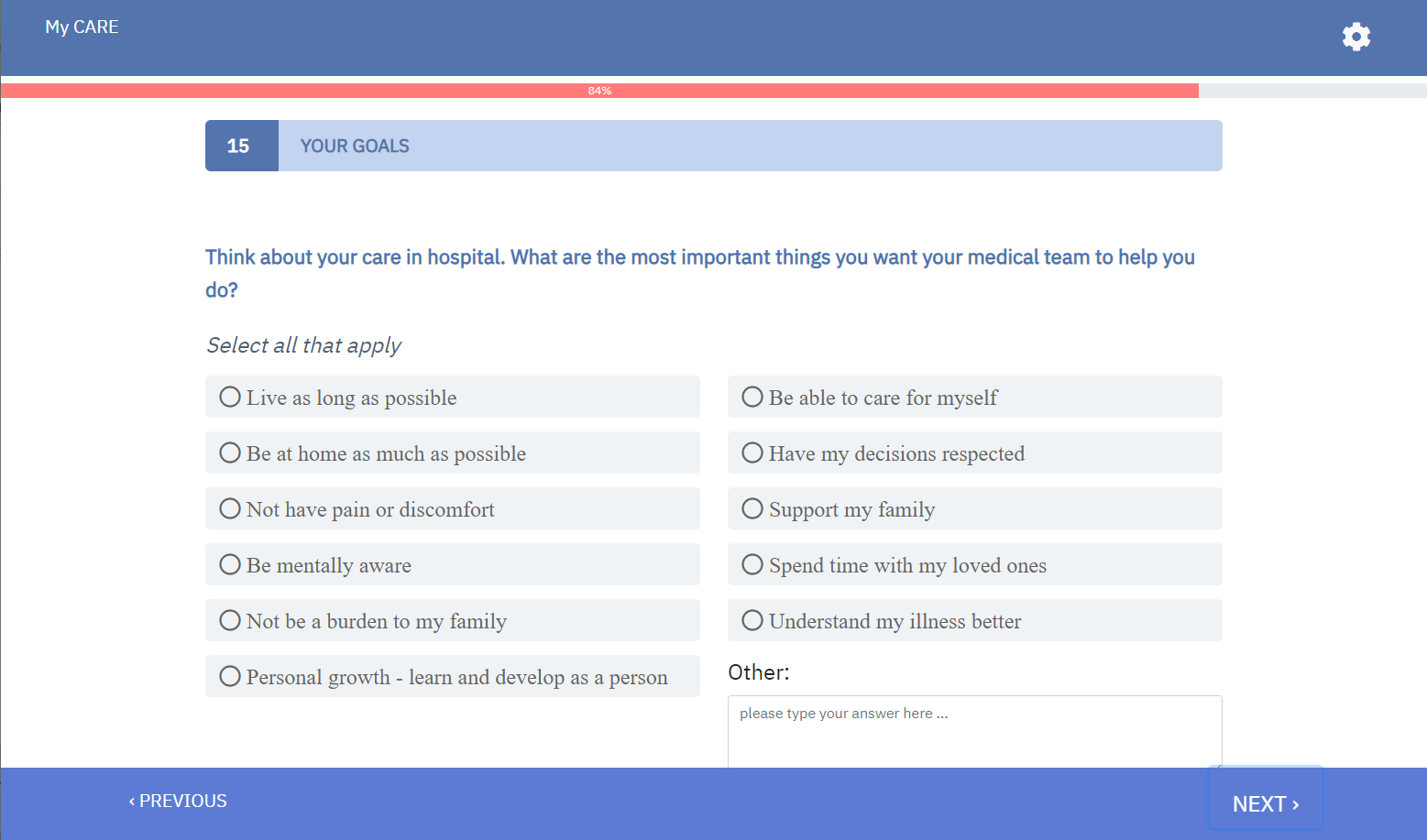


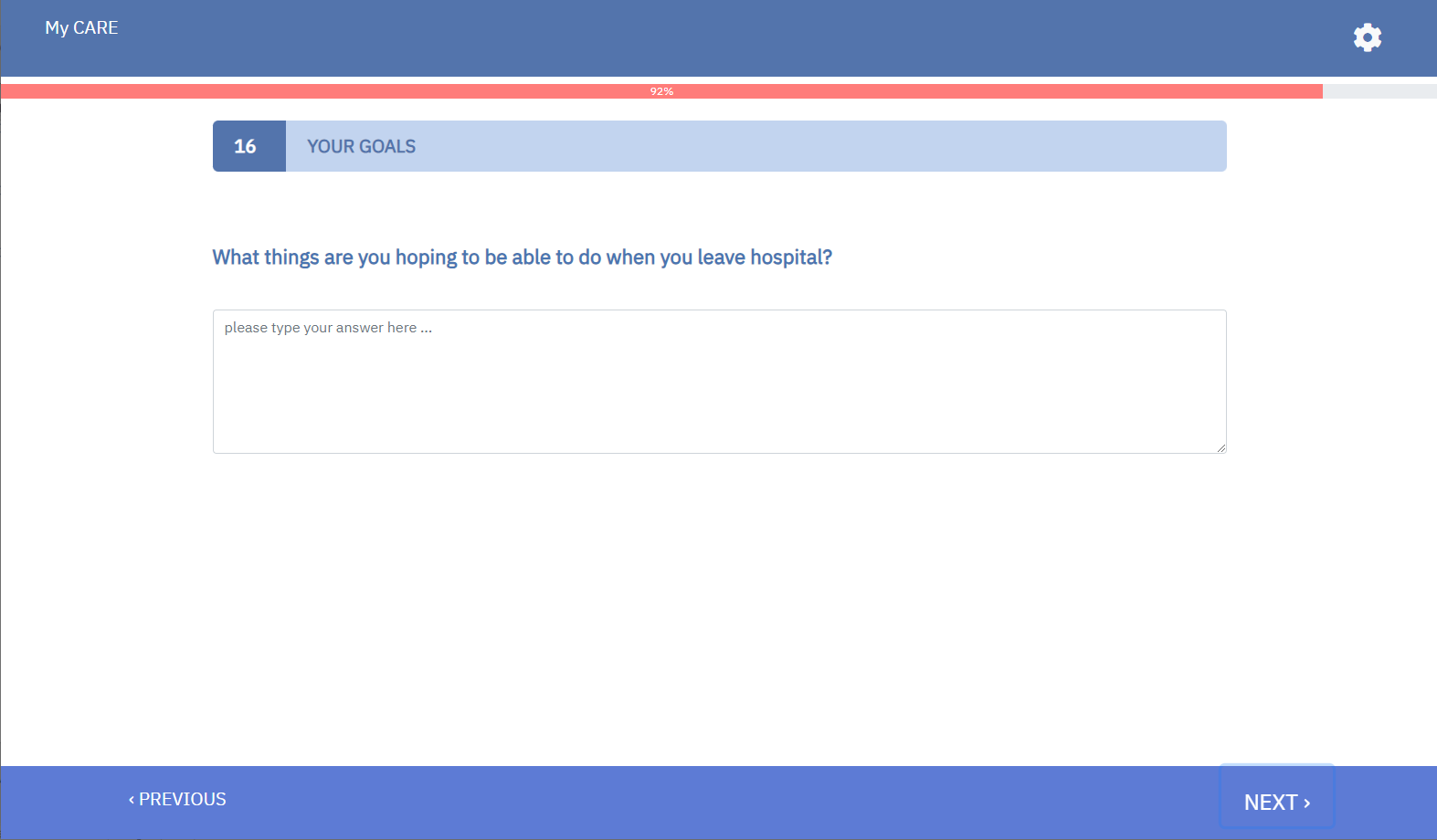


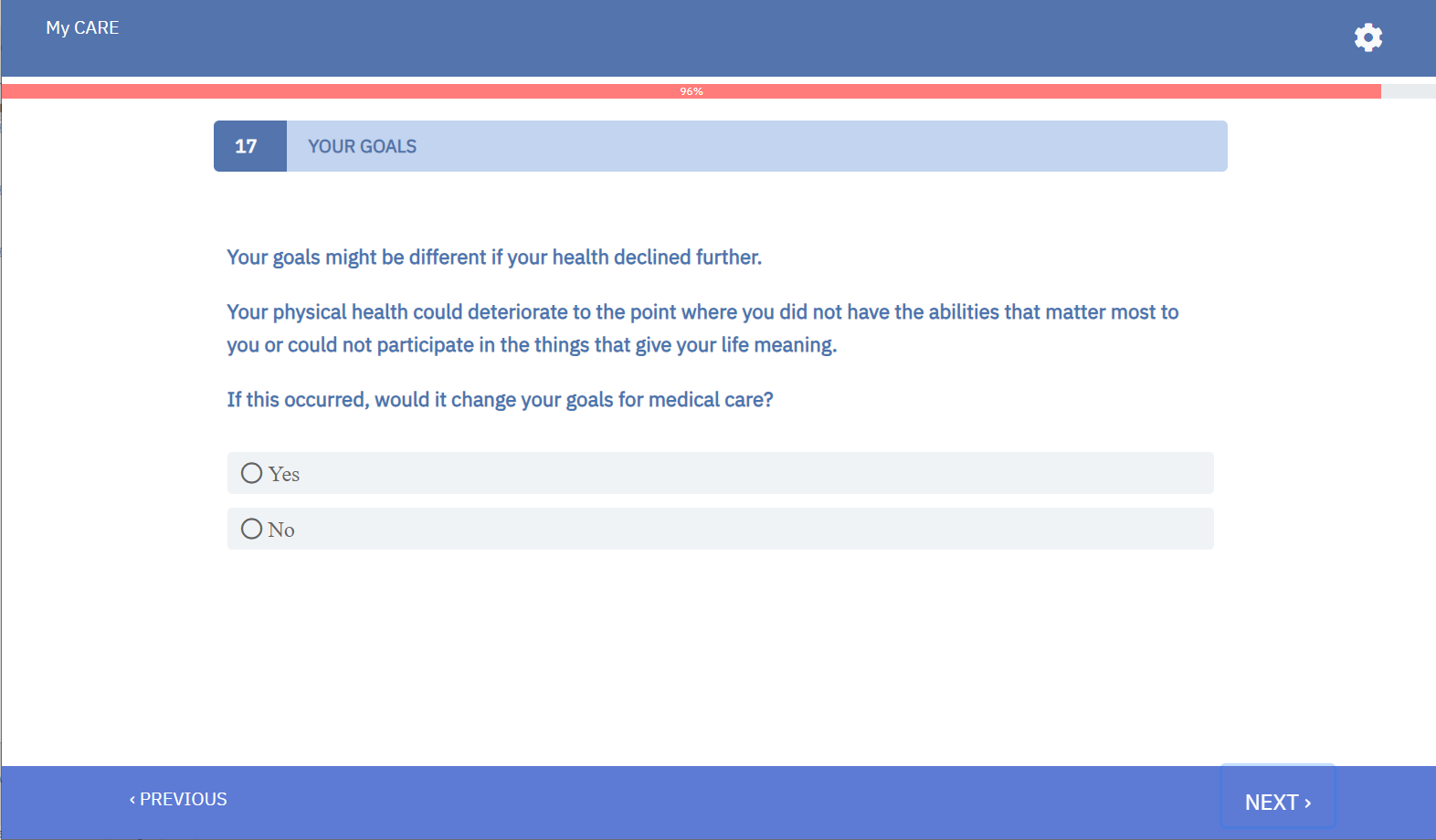


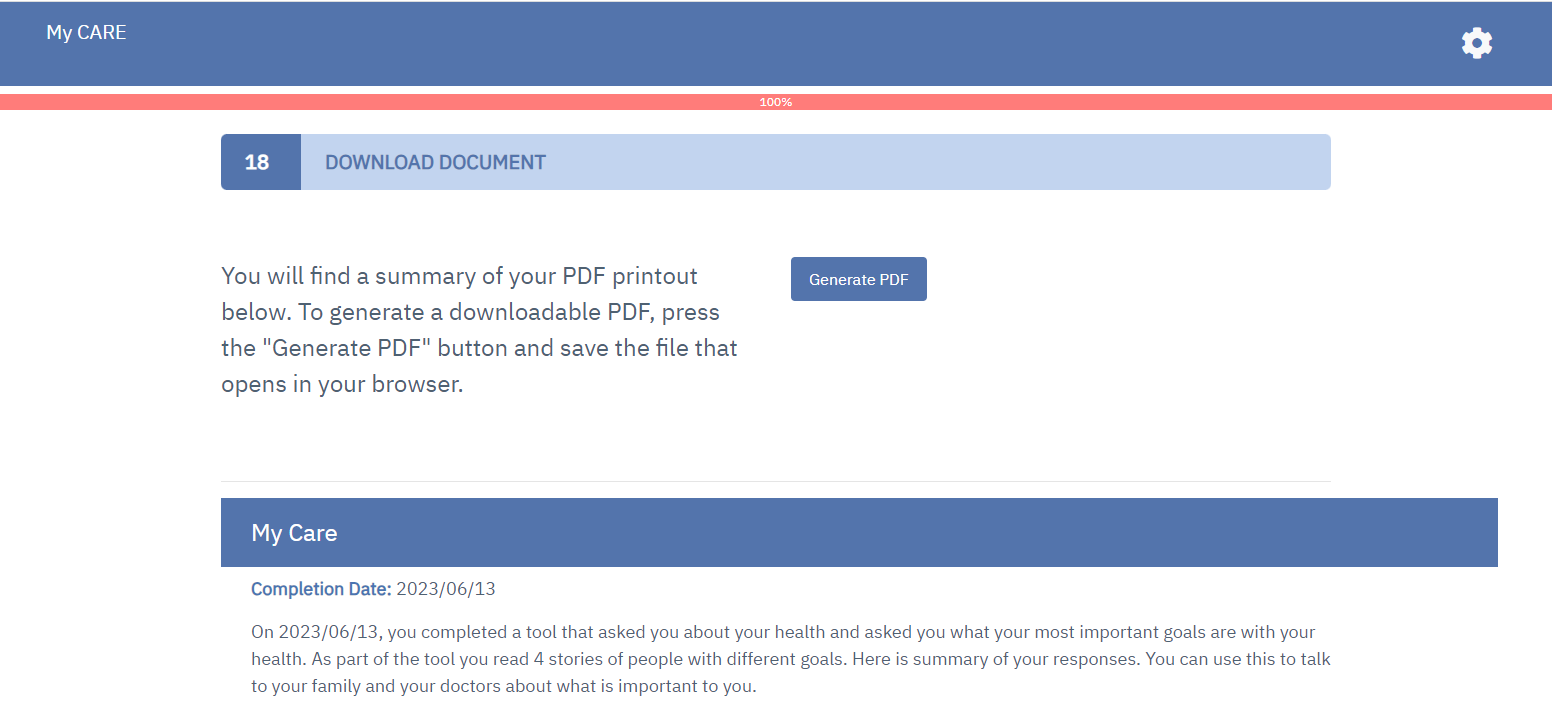

Supplement: Multimedia Appendix 2 [file formative_v9i1e66932_app2.docx]
