# Supplementary material for: Evaluation of a Novel Goals-of-Care Discussion Priming Tool (MyCare) in Inpatient General Internal Medicine Ward Settings: Feasibility, Acceptability, and Usability Study
Source: JMIR Form Res. 2025 Oct 28;9:e66932. doi: 10.2196/66932 (PMC12605267; doi:10.2196/66932)
Supplement: Multimedia Appendix 3 [file formative_v9i1e66932_app3.docx]

**Appendix 3:** Semi-Structured Interview Guide for Patients

Can you tell me about using the tool?

Were you able to complete the tool?

How did you feel when using the tool?

Prompts: For example were you upset, worried, relieved, satisfied or interested?

Were there specific questions or topics that promoted those feelings?

What parts of the tool were most useful?

Can you tell me more about that?

How did it help you?

What parts of the tool were least useful and could be removed?

Why?

Is there anything that could be added to the tool to make it better?

What did you learn from using the tool?

Can you explain how what is most important to you can help you make a plan of care together with your doctor?

If not already discussed:

Did the tool help you think about and understand what is important to you?

Did the tool help you think about the kind of care you would want if you became more sick?

Is the document you received after using the tool useful?

How do you plan on using the document you were given?

Do you have any other thoughts or comments?
