# Supplementary material for: Evaluation of a Novel Goals-of-Care Discussion Priming Tool (MyCare) in Inpatient General Internal Medicine Ward Settings: Feasibility, Acceptability, and Usability Study
Source: JMIR Form Res. 2025 Oct 28;9:e66932. doi: 10.2196/66932 (PMC12605267; doi:10.2196/66932)
Supplement: Multimedia Appendix 4 [file formative_v9i1e66932_app4.docx]

**Appendix 4:** Themes from Patient Interviews:

| **Patient Interview Themes** | | | |
| --- | --- | --- | --- |
| **Theme** | **Subtheme** | **Code** | **Example(s)** |
| **Usability** | **Motivators for tool use** | **The tool was easy to use:**  Users found the tool easy to use independently or after initial guidance on how to use an iPad | “[The tool] is not at all complicated. It does need a bit of guidance to start with and then you know what’s going on afterwards but it's certainly not complicated.”  “I found it really directive. So that it helped you make decisions.” |
|  |  | **The tool was enjoyable to use:**  Users found the tool likeable, fun or interesting and felt confident or relaxed while using it. | “Oh it was good, it was fun actually. It was very interesting.”  “As far as I’m concerned it's probably a great tool and I would probably use it.” |
|  |  | **Using the tool made me feel empowered towards making decisions** | “I felt really gah, I don’t have the word. Like not smart, but kinda like as if I’m not the stupid about the whole thing and so that to me was uplifting. Yeah empowered”  “yeah it did. I can’t say whether it was a good feeling but it made me think well I can change if I don’t like that feeling possibly. I can go to my doctor and say okay well how can I make this be the way I want to do it myself.” |
|  | **Barriers to tool use** | **Continued support was needed to complete the tool:**  Ongoing support was needed from the RA to use the tool or iPad | “Well [using the tool] was okay with your help but by myself I would have been stuck.”  [Were you able to complete the tool?]  “With a lot of help yes” |
|  |  | **The tool was difficult to use:**  Users found the questions difficult to answer or the iPad difficult to use | “I felt I kinda had to struggle to get the right answer and put myself in the position the author wanted me to be in. I found it difficult.”  “I find it rather awkward to complete. And some [questions] required quite a bit more knowledge than I had.” |
|  |  | **The tool was too repetitive:**  Users felt the tool was asking the same questions in different ways | “Frustrating in that you repeat the question over and over again and I don’t know. That’s kind of my opinion on using the tool”  “ Well some were repetitive. So if they are repetitive they may not need to be stated because they were basically stated the same way or could be stated in a different way to look for a different perspective on it.” |
|  |  | **The tool was too black and white and needs space for flexibility as things change:**  Users thought that answer choices in the tool were too black and white and did not reflect their opinions or did not capture how opinions may change as disease progresses. | “A lot of the questions are yes or no answers. But a lot of them are not yes or no answers, they are in between. Or there is an explanation to the yes and the no and that is not provided. I think is should say yes and qualify, or no and qualify that way you get the answers down that I gave you afterwards. Because that aside it could be 100% yes or it could be in that grey area. You don’t know.”  “The way you interpreted the question was dependent on who you’re referring to. And the other thing is as your disease progresses and it changes, your opinion is going to change. It’s all of those that [the tool] need to be oriented towards. Like if you heard you were going to die in the next two weeks are you prepared to do this are you prepared to do that, have you talked to your doctor? Like we have prepared in our minds, but I am not going to die in two weeks, so I have a totally different perspective on that but there didn’t seem to be any give and take or flexibility in that, where you are in the progress of your disease. |
| **Perceived Usefulness** | **Reflection** | **The tool was informative:** | “Its very informative. It gives you an awareness” |
|  |  | **The tool helped me think about what decisions I may need to make in the future:**  The tool prompted users to start thinking about what is important to them and future health decisions. | “Uhm a couple of questions made me think a little bit. But they are all necessary to know. And I had to think a bit. So, in that respect if you haven’t already thought of it should stimulate you to think ‘I should start.’”  “I think maybe people like this uh we don’t think about a lot of questions you raised and makes me think more about what lies ahead” |
|  |  | **The tool helped me understand myself better and clarify what is important to me:**  Users felt that using the tool helped them understand their personal priorities and understand what is important to their care. | “It clarifies. It helps to clarify once you understand your personal priorities you can plan your actions based on that.”  “It made me think about what I really want. And I want to get back to normal. I may not be able to, but I want to get back as close as possible.” |
|  |  | **The content of the tool related to my experience:**  Users found the content of the tool was familiar and they were already thinking about the included topics | “Yes. I’ve already thought about [what’s important to me] before but it reinforced my way of thinking”  “ And the questions that were asked no doubt had popped in my mind as well as others so all I would find out of it is ‘I did think about this’ or ‘I thought about that.’” |
|  |  | **The content or modality of the tool did not relate to me:**  Users found the tool content did not reflect them as patients or thought the content of the tool would be better delivered in a different way. | “Yeah I mean if you give it to your doctor you could make a plan of care but I don’t know why you just can’t go talk to them.”  “Well I don’t relate to any of the stories” |
|  |  | **I plan to use the document for my own records or reflection:** | [Is this document useful?]  Patient: “Oh for my own personal perhaps. “  Caregiver: “Well I think you want to make sure you have the proper things in place for your wishes to be catered.”  Patient: “Yeah”  [Is this document useful?]  “to help me reflect on what I said and how I thought when I was answering the questions” |
|  |  | **I learned nothing from the tool:**  Users felt they learned nothing from the tool or the tool was familiar to conversations they previously had. | “I can’t think of anything specific I learned no”  [What did you learn from using the tool?]  “No. Did it serve as a decent review? Yes. Learn and review are closely related but different” |
|  | **Sharing communication** | **I plan to share this document with my care team and/or the people who matter to me**: | [Is this document useful?]  “To me no, but it might be to the family to show it to you know the kids and give them an understanding of how I feel without having to say. I mean they might not agree with it, but it will tell them where I stand.”  “Eventually at sometime I should mention it to my GP” |
|  |  | **The tool reinforced that I have been doing and asking my doctors the right things:** | “I learned that uh a lot off the things I have been thinking and talking to doctors about has been the right thing to do”  [What did you learn from using the tool?]  “Just that I’m basically doing what I’m supposed to do.” |
|  |  | **Knowing what’s important to me can help me talk to my doctor about what I want:**  Users expressed that using the tool and knowing what was important to them would help them talk with their doctors about the type of care they want. | “ I think the doctor can clarify what lies ahead for me that I don’t realize right now. I think I just walk the path without asking why and I think the doctors can very well help me understand why I’m going through certain steps” |
|  | **Provider expectations** | **Doctors need to be willing to listen and act for this tool to be useful:** | “Providing the doctors are able and willing to listen and would take action to what I’m saying. Because some doctors just ‘whoops ahahah’ giggle it off and I cannot stand that. It's like what you are saying, it's alright you know. But listen as we get older that’s part of life and we are all getting older.”  [Is the document useful?]  “No... well it depends on the doctors you have to deal with” |
| **Emotions** |  | **Using the tool brought up negative feelings:**  Using the tool brought up negative feelings such as being tired, confused or worried. | “ [ I felt] worried about ‘how much do you want to know’ I am not sure. But who would I talk to? It would be more my friends I would talk to before anything.”  [ how did the tool make you feel?]  “Tired. Yeah to be honest with you. “ |
|  |  | **The tool did not prompt strong feelings:**  Users expressed neutral feelings towards using the tool. | “No. I guess questions are questions. I was being objective. I wasn’t being emotional in any way.”  [How did you feel when using the tool?]  “Indifferent” |
