# Supplementary material for: Evaluation of a Novel Goals-of-Care Discussion Priming Tool (MyCare) in Inpatient General Internal Medicine Ward Settings: Feasibility, Acceptability, and Usability Study
Source: JMIR Form Res. 2025 Oct 28;9:e66932. doi: 10.2196/66932 (PMC12605267; doi:10.2196/66932)
Supplement: Multimedia Appendix 5 [file formative_v9i1e66932_app5.docx]

| **HCP Interview Themes** | | | |
| --- | --- | --- | --- |
| **Theme** | **Subtheme** | **Code/Definition** | **Example(s)** |
| **Usability** | Facilitators and Barriers | **The tool would help facilitate goals of care conversations under time constraints:**  Time constraints pose as a barrier towards having goals of care conversations. The tool helps elicit important background information that can motivate health care providers to talk to their patient and provides direction for a goals of care discussion. | “Part of the challenge while on service is not enough time to do the necessary background, you know, getting to know the patient. So this helps you get a step ahead and gives you the information available to then have the conversations . . . I would use this information because it kind of helps with gathering the necessary context and background so that I'm more efficient when I have a goals of care conversation. If I saw this on a patient’s chart, I would read it, I would get to know them, and then it's still important to go through some of the questions when you talk to the patient, but it will give me a more nuanced understanding of where they're coming from.” |
|  |  | **I would be more motivated to use the tool if it was supported on an institutional level.** | “Will the hospital back it? So, let's say you know it as it does frequently happen where you know you're trying to do what's best for the patient, you pull out this document and you say, well, look, you know, this Mrs. Smith clearly all she wanted to do was just to live until February. So, I'm, you know, granting her request. We have thought of care involved and then the entire family is like ‘what do you mean? I just talked to my grandmother last week and she's fit as a fiddle and she's talking about bingo and you know, I don't believe that document you guys are all in a ring together and this is conspiracy and you better, you know, keep her alive or else.’ And will the hospital back you? Because what we've encountered in the past is that the hospital will always err on the side of saying, well, actually we should we should bring in a mediator, which typically means lawyers, right, and then the process gets dragged down and so on and so forth.” |
|  |  | **The tool will be useful for some patients but not others** | “Yes, I think it depends on who I received the report for. If I received the report for someone who I am looking to do a more in depth goals of care discussion, you know, be it because they come from either a long-term care home and I feel that maybe the goals of care need to be readdressed because now they have a new illness, or if they've had recurrent admissions to hospital where I think their trajectory over the past year kind of shows me that they may not do well in the upcoming months or year. Then I think in that setting it would be really helpful. So I think it would be somebody who I have identified as potentially needing a serious illness conversation” |
|  |  | **I would trust the tool more if patients updated their responses frequently.** | “I would always want to verify [the results]. It's almost like I would want to do it again because I don't trust it. Maybe that's because it's new, right? Maybe we do need to get to the point where we say, okay, you know what this was done in November and now it's January and so we should trust this and not have to reinvent the wheel again because I think as physicians we are always a little bit hesitant to trust something written down months ago and take that like ‘are you sure you still feel that way, right?’” |
|  |  | **The tool needs to be used at the right time during patient care for it to be helpful.** | “The question is how? I think that that's the thing, and like when? ‘Cause sometimes, you know when people come in and they're like super, super sick, this is not the time to do this and then, you know, like the timing of when we get the information. I'm not sure how that plays out exactly from an implementation point of view. There's probably a sweet spot, right? Like when they're stable and you're planning discharge as opposed to you don't want this to be on the day of discharge where you would be like by the way, let's clarify your goals of care. You wanna still be able to have kind of that trust building and that relationship with [the patient]” |
|  |  | **Usability of the tool would depend on how readily available the results are to the physician** | “I think [whether I would use the tool] is gonna just really be the timing and getting it to [the doctor], right? Like I think that those would be the big things.” |
|  |  | **Time constraints may prevent me from reviewing patient’s responses to the tool.** | “I think [I would use the tool] definitely if there's time. I think that's the main limiting factor. It takes some time to kind of process these documents and use them in a discussion with the family and patients. So, I think to me it's highly variable depending on how busy and how much staff you have on the CTU. Because like I acknowledged I got one . . . but like it was just crazy that week. I didn't have the time to sit down and kind of plan a meeting and so you know, it takes it takes some time to kind of process these documents and use them in a in a discussion with the family and patients. So, I think to me it's highly variable depending on how busy and how much staff you have on the CTU.” |
|  |  | **For the tool to be useful, patients need to understand how to use it to talk with their family and healthcare team** | “[…] they need to have better understanding of why they're being sent this. So that first introduction paragraph I think could be beefed up a little bit . . . I don't think patients have a clear idea usually of how this kind of information can be used to speak to their own family members or to the physicians who are admitting them to the hospital because it's not something that's talked about in the general discussion, right?” |
|  |  | **Would be more usable if it could be used with patients who don't speak English as a primary language** | “Sometimes it's more intuitive if English is their first language, but if they can fill this out when English is not their first language, I suspect that we'll be able to pick up some stuff.” |
| **Perceived Usefulness** | Guiding conversations | **The tool can serve as a framework for having a goals of care conversation**  This code captures the value of having in a framework across two settings: 1.) a framework to reduce individual bias in GOCD, and 2.) for training purposes with junior staff and trainees. | “I think [the tool] would mostly be helpful to help us frame the discussion. I think we often make assumptions about what we would want for ourselves and we frame the discussion that way, and I think that probably leads to a lot of miscommunication if we knew upfront what mattered most to people, I think we would be better able to convey our concerns about what that might look like in the setting of illness.” |
|  |  | **The questions asked in the tool serve as good prompts for initiating goals of care conversations** | “So when you know ‘what do you want to do when you leave the hospital’, you can use that as a springboard to really get into, you know, someone says to me, ‘I want to be able to care for myself and I want to spend time with my family’. Okay, well, that's a springboard for having a conversation with you. ‘I don't think we can fix these things. I think this is going to deteriorate. You know, doing these treatments will make you suffer and won't achieve those goals. So, putting you on a respirator or doing CPR.’ So, these are good springboards to having those difficult conversations. But it's, but it's simplified, right? It's simple.” |
|  |  | **The tool helps elicit information to help health care providers and patients get on the same page** | “Ah so as I mentioned starting the conservation if they haven’t had it or thought about it before and then eliciting everything that you guys have there. So, eliciting their understanding of their illness, what their baseline function was and any current symptom burden in an objective way, which I think is super helpful, and then what matters most to them. And I love that there's three stories, right. I think 3 versions. I like that because I think people can try to identify with each of those stories and then that can help us kind of align what they think they understand with what their disease is and kind of how to move forward.” |
|  | Reflection: | **The tool promotes reflection and can be used as a primer for both patient and physicians before having a goals of care conversation.** | “ I think what [the tool] does is it really sets the stage for understanding what [the patient’s] values are. So I definitely think you know, getting it before I had a goals of care discussion with the patient would be really helpful. I think for me to understand where that coming from, but also even for them to start getting them thinking about these things.”  “The [tool is helpful] to know [patients] have thought about it before. Talking to so many people, you get the impression that they've never thought about [goals of care] before. They just are content to just continue existing until a crisis hits and then they're forced to make some drastic decisions, but they're probably not equipped to make those decisions or not in the best state of mind or health to make those decisions when they most need it” |
|  | Shared decision making | **The information patients share in the tool would be useful for the entire healthcare team to have.** | “I think it’s a good. It's actually very useful to have examples patients can look up and compare with their own experience. That would be, that’s one important tool in empowering the patients and families more. So even just sharing this with them after a preliminary conversation, I think it would get them more engaged. But also, from the provider’s side of thing, if its available not just for the physicians but also the other healthcare workers that would be also helpful.” |
|  |  | **The tool helps build confidence that doctors are making decisions that align with their patients’ wishes** | “Those goals always get made. Just, you know, the confidence of which you've made them right. Whether you've talked to enough family or whether or not it’s the first time you talk to Mr. Smith. Was he truly with it or not so much, right? And then the family come and they doubt your word. And so ‘ohh, my father would have never said that’ and all that kind of stuff. So having extra information is always very helpful. Positive or negative.” |
