# Supplementary material for: Evaluation of a Novel Goals-of-Care Discussion Priming Tool (MyCare) in Inpatient General Internal Medicine Ward Settings: Feasibility, Acceptability, and Usability Study
Source: JMIR Form Res. 2025 Oct 28;9:e66932. doi: 10.2196/66932 (PMC12605267; doi:10.2196/66932)
Supplement: Multimedia Appendix 6 [file formative_v9i1e66932_app6.docx]

**Appendix 6:** Suggested Changes Summary

**What parts of the tool were the most useful?**

| **Response** | **(n)** | **Example(s)** |
| --- | --- | --- |
| All of it was useful | 5 |  |
| Asking about my goals for when I leave hospital and what I think will happen in the future | 5 | “it made me think about what I really want. And I want to get back to normal. I may not be able to but I want to get back as close as possible.” |
| Asking me what type of care I would want near the end of life | 4 | “more about your power of attorney your spouse and family and how far do you want to go. You know not everybody wants to fight” |
| Identifying people who are important to me | 4 | “the fact that you are gathering information on who is important to me” |
| The structure of the tool.   - Having pre-specified answers to choose from - Being able to enlarge text | 3 | “Having the answers you had to choose from, if you had to get something from yourself it would be harder” |
| Asking about what is important to me and what wishes I want to be respected. | 3 | “Oh what’s most important to me it asked. Which I thought was a good question because some people won’t take that into consideration. They are thinking of themselves rather than other people and what they think is best. But they should really think about what the person wanted. That’s one thing.”  “well there was things like are you going to ask your health professional to respect your wishes and do thing that way instead of any other way. That was the thing I found interesting. Yeah because I already decided I don’t want to be lying around with a crushed chest or no quality of life” |
| Asking if my goals would change if I became more sick | 1 |  |
| Asking about my priorities | 1 | “Most of them were useful. But the part about getting older and close to death and prioritizing and reflecting on the things that made me aware to do the things I am more aware of.” |
| Being able to have input on what doctors might talk to you about | 1 | “I thought it was useful to think you are having little influence in what the doctors might communicate with you about.” |

**What parts of the tool were least useful or could be removed?**

| **Response** | **n** | **Example(s)** |
| --- | --- | --- |
| Nothing | 8 |  |
| The tool was too repetitive | 3 |  |
| The stories had too much overlap which made them hard to choose from | 1 |  |
| Asking what I think about my illness | 1 | **“**P: The one that says answer what best describes what you are thinking about your illness.  R: You think that could be removed?  P: Yeah that could  R: Is there a reason why that wasn’t useful to you?  P: I don’t know. I thought oh not again.” |
| Asking who supports me was not clear because different people support me at different times and in different ways. | 1 | “If you have a conversation about your care who would you like to be there. I put siblings because my parents are gone and so they would be the most important. But there are friends and people who have helped me considerably along the way and I wouldn’t want them to feel slighted. Although with siblings, they are the ones in the will more than others so I guess that has to be taken into consideration. That’s one thing. And who are the people in your life who help and support you? At different times its always different people. I live in a retirement home now. I know people I didn’t know before who are very good. I am good to them and they are good to me. So I would like to think of them as being okay as well” |
| The iPad | 1 |  |
| The question that asks you to narrow your list of what’s important to you to answer | 1 |  |

**Is there anything that could be added to make the tool better?**

| **Response** | **n** | **Example(s)** |
| --- | --- | --- |
| Nothing | 7 |  |
| More on mental health | 2 | “That’s your right that’s another good point. That’s a big chunk of the specific of what I was saying about clinical parameters versus quality of life. Mental health should be front and center in terms of that. I mean it’s the most important aspect of quality of life.” |
| Questions about medical assistance in dying | 2 | “how much knowledge do you have about things like homecare uh things like MAiD. That’s the thing where you might want to add in a question or two. Uhm that’s very much a question yup.”  “I’m not whether you would even want to do it. But whether you would want to put down a question of medically assisted suicide. I don’t know whether you would want to put on or not. I wouldn’t want to for myself. I think too many people are asking for it. So I’m not sure I would want that question raised, because I would not want to be alerted to it. You go around it and talk about end of life care.” |
| Awareness of nature and thanking god | 1 | “yeah the awareness of nature and thanking god for what we got.” |
| Asking about how much knowledge people have about homecare or community care alternatives | 2 | “how much knowledge do you have about things like homecare”  “Well for people who are long term care knowing the community health alternatives is important.” |
| Adding space where I can elaborate or add comments about my answers | 1 |  |
| The tool is long enough already | 1 |  |
| More clarification on “how much do you want to know about your illness?” ex. How much do you already know? | 1 | “ The question asking ‘how much do you want to know about your illness?’ bothers me a little bit because it is very abstract and vague. I don’t know what you would do about that … I think for that to be useful there needs to be more specifics within. Some examples, something because otherwise you know the doctor who is getting the results goes this person wants to know more. How much does this person know? What’s more? So you need to work on that one I think.” |
| Putting the tool on a website people can access at home while healthy. | 1 | “But if this is something that is a link that goes on a site and that people want to know more information not about the tool but about the health care I think this would be great. To have a tool or cloud where you can just go click and find more information. I liked the little stories those were helpful. So yeah it would be helpful to have something on a website. I don’t know where this belongs but yeah… seeing this while I was healthy is just as important” |
